# Synthesis of Highly Ordered Amphiphilic Polymer Conetwork Hydrogels via the Topologically Precise Interconnection of Two Highly Incompatible Polymers

**DOI:** 10.1021/acs.chemmater.5c00127

**Published:** 2025-07-24

**Authors:** Demetris E. Apostolides, George Michael, Konstantinos Andronikou, Costas S. Patrickios, Szabolcs Pásztor, Györgyi Szarka, Anna Petróczy, Béla Iván, Takamasa Sakai, Sylvain Prévost, Dimitrios G. Tsalikis, Michael Gradzielski

**Affiliations:** † Department of Chemistry, 54557 University of Cyprus, P.O. Box 20537, 1678 Nicosia, Cyprus; ‡ Polymer Chemistry and Physics Research Group, Institute of Materials and Environmental Chemistry, Hungarian Research Network, 280964 Research Centre for Natural Sciences, Magyar tudósok körútja 2, H-1117 Budapest, Hungary; § Department of Bioengineering, Graduate School of Engineering, 13143 The University of Tokyo, 7-3-1 Hongo, Bunkyo-ku, Tokyo 113-8656, Japan; ∥ 56053 Institut Max von Laue—Paul Langevin (ILL), 71, avenue des Martyrs—CS 20156, 38042 Grenoble cedex 9, France; ⊥ Particle Technology Laboratory, Department of Mechanical and Process Engineering, ETH, Zurich CH 8092, Switzerland; # Stranski-Laboratorium für Physikalische und Theoretische Chemie, Institut für Chemie, Straße des 17. Juni 124, 26524 Technische Universität Berlin, D-10623 Berlin, Germany

## Abstract

Here, hydrophobic polyisobutylene and hydrophilic poly­(ethylene glycol), both of reasonably high molar masses, have been end-linked, yielding amphiphilic polymer conetwork (APCN) hydrogels that can self-organize in water into well-ordered lamellar structures. The cross-linking of hydrophobic and hydrophilic polymer segments produces networks that typically exhibit sphere-like nanodomains in water and in the bulk, but the orderly interconnection of relatively large and highly incompatible polymers leads to hydrogels that internally assemble into lamellae. This unprecedented result may be attributed to the weak force-field established by the presence of a minimal concentration of homogeneously distributed cross-links in the case of the present system, which must be contrasted to a higher concentration of randomly placed cross-linking points, which destroy long-range ordering in conventional APCN hydrogels. Significantly, the presently developed APCN hydrogels maintain good tensile mechanical properties, with their strain-at-break reaching a value of 800%. This study puts forward the design concepts for attaining highly ordered hydrogels, which would confer upon them better transport and mechanical properties and broaden their utility in biomedical and energy applications.

## Graphical abstract

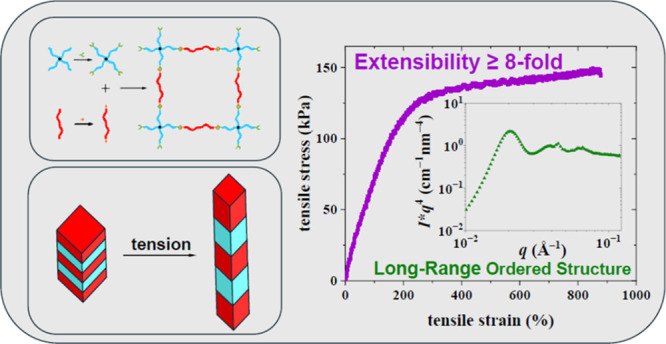

## Introduction

Polymeric hydrogels consist of cross-linked polymers and water, with the latter component representing the main ingredient, and with the mass ratio of water-to-polymer usually being in the tens, hundreds or even thousands range.^1–3^ Although very useful in biomedical and technological applications, these materials have been suffering from fragility.^4^ Intense research efforts during the past 25 years have partially addressed the fragility problem by developing hydrogels possessing enhanced mechanical properties compared to conventional counterparts. These efforts initially resulted in the development of rotaxane-linked networks,^5^ nanocomposite networks,^6^ and double-networks.^7^ Later efforts led to the preparation of ionically linked (polyzwitterionic) networks^8^ and entanglement-rich networks.^9^

Block copolymers^10,11^ are important contemporary materials finding applications as high-performance (thermoplastic) elastomers (e.g., Kraton) with enhanced thermal and chemical stability as compared to conventional rubber, materials for energy-saving water separating membranes (e.g., NEXAR), biocompatible coatings on coronary stents (e.g., the Taxus coronary stent whose coating also delivers a preloaded drug) or pacemakers, and advanced surfactants, whereas emerging block copolymer applications include high-stiffness/high ion-conductivity polymeric electrolyte membranes for batteries, ion gels that enable fast switching of organic transistors, and poly­(ionic liquid)-based membranes for electrochemical applications and gate dielectrics.^12–14^ All of the above applications rely on the property of block copolymers to self-assemble on the nanoscale in the bulk^12–14^ or in solution.^11,15,16^

Cross-linking (linear) block copolymers can convert them to elastomers, thermosets, gels^17,18^ or even hydrogels, if one of the blocks is hydrophilic. Furthermore, if the other block is hydrophobic, the resulting hydrogels, which are now based on cross-linked amphiphilic block copolymers, a type of amphiphilic polymer conetworks (APCNs), are mechanically robust materials, thereby offering another yet option for strong hydrogels.^19–21^ This mechanical robustness arises from their lower aqueous swelling due to the presence of the hydrophobic component, and also to their self-assembly, as the formed hydrophobic domains reversibly dissipate a large amount of mechanical energy upon their deformation.^22–24^ However, the constraints introduced by the cross-links may have a negative impact on the quality of the self-assembly in these block copolymer-based hydrogels, hampering an orderly nanophase separation, leading to structures possessing only short-range order.

To date, the design and preparation of hydrogels with highly ordered structure has been given much less attention as compared to making hydrogels mechanically robust.^25–29^ Although a highly ordered structure may also confer upon the hydrogel better mechanical properties, there may be further benefits arising from this orderly segregation. These benefits concern gel applications related to solute transport, such as drug delivery^30^ and salt diffusion in polymer electrolytes for lithium-ion batteries,^31^ so as to guide solute migration in the desired direction, or even block it altogether at will, provided that one of the (continuous) nanophases is completely impermeable by the solute. Thus, a timely objective would be to develop APCNs^32–37^ based on cross-linked block copolymers, with APCNs self-assembling on the nanoscale with long-range ordering, yielding the well-defined morphologies formed by their non-cross-linked precursors. The feasibility of this objective has recently been proven in a dissipative particle dynamics (DPD) simulations study, which indicated that model APCNs based on end-linked block copolymers essentially form the same ordered morphologies as their free block copolymer precursors, i.e., lamellae, cylinders, spheres.^38^

Despite the findings of the above-mentioned simulations study, most experimental investigations on APCNs based on cross-linked block copolymers and other segmented architectures attest to APCN nanophase separation with only short-range order. In particular, most recorded transmission or scanning electron micrographs (TEM or SEM) and small-angle X-ray or neutron scattering (SAXS or SANS) profiles exhibit blurred spheroidal structures, and single and usually broad correlation peaks, respectively.^39–51^ The few studies on APCNs nanophase separating into structures with long-range order appear to share four common characteristics.^52–54^ The first one is the use of a minimal amount of cross-linker placed in an orderly manner, at the chain ends. The second one is the employment of well-defined polymeric building blocks, such as star polymers and block copolymers. A third characteristic is the larger size (molar mass) of these building blocks to facilitate nanophase separation and its identification, whereas a final desirable characteristic is the softness of both the hydrophilic and hydrophobic components to reduce morphology equilibration time.

In this study, we pursue the development of mechanically robust APCN hydrogels with block copolymer structure, exhibiting much improved nanophase separation with long-range ordering. To this end, the concepts outlined in the previous paragraph are adopted. In particular, well-defined polymeric building blocks of reasonably high molar mass, from 5 to 20 kDa, are employed, interconnected at their termini via just one linking point. Furthermore, the hydrophobic component is very soft and amorphous, with a very low glass transition temperature, whereas the semicrystalline hydrophilic component softens up upon hydration. The former component is polyisobutylene (PIB),^55^ whereas the latter is poly­(ethylene glycol) (PEG).^56^ The fact that PIB and PEG are highly incompatible to each other^57,58^ provides an extra characteristic that may further assist in the long-range ordering of the APCN self-assembled structure. The developed APCNs are highly extensible, with the best sample possessing a strain at break of 800%. Most importantly, thermally annealed APCNs self-assemble in D_2_O to yield lamellae with long-range order (two higher-order peaks visible) instead of the spheroidal structures typically exhibited by APCNs.

## Experimental Section and Simulations/Calculations

### Materials

4-Formylbenzoic acid (97%), *N,N’*-dicyclohexylcarbodiimide (99%), 4-dimethylaminopyridine (≥99%), calcium hydride (CaH_2_, 90–95%), glacial acetic acid, *N*,*N*-dimethylformamide (DMF, 99.8%), benzene (≥99.0%), tetrahydrofuran (THF, ≥99.5%), deuterated benzene (C_6_D_6_, benzene-*d*_6_, 99.5%), deuterated chloroform (CDCl_3_, 99.8%), and dialysis tubing composed of benzoylated cellulose with molecular weight cutoff of 3000 g mol^–1^ (average flat width 32 mm) were purchased from Sigma-Aldrich-Merck, Germany. *N*,*N*,*N′*,*N′*-Tetramethylethylenediamine (TMEDA, 99.5%) and allyltrimethylsilane (ATMS, 98%) were purchased from Sigma-Aldrich, St. Louis, Missouri, USA), whereas *n*-hexane and methanol were obtained from Molar Chemicals, Halásztelek, Hungary. Tetrahydroxy-terminated four-armed star poly­(ethylene glycol) (tetraPEG star) of number-average molar mass (*M*_n_) of 5000 g mol^–1^ (tetraPEG-5k) was purchased from JenKem (Plano, Texas, USA), whereas tetraPEG stars with *M*_n_ values of 10,000 and 20,000 g mol^–1^ (tetraPEG-10k and tetraPEG-20k) were purchased from NOF Corporation, Tokyo, Japan. All three above-mentioned tetraPEG stars were modified in a three-step procedure so as to convert their hydroxyl end-groups to benzaacylhydrazide ones and obtain tetraPEG-Hz, according to previously published work.^45,59,60^ Isobutylene (IB) was from Messer Hungarogáz Kft., Budapest, Hungary. The bifunctional initiator for the quasiliving carbocationic polymerization (QLCCP) of IB, *tert*-butyldicumyl chloride, was prepared in three steps, following a procedure described earlier.^61,62^ α,ω-Dibenzaldehyde-polyisobutylene (Bz-PIB-Bz) was prepared after a series of end-group modifications of α,ω-dichloro-polyisobutylene (Cl-PIB-Cl) that had been synthesized from the QLCCP of IB using *tert*-butyldicumyl chloride.

### Syntheses

#### Synthesis of α,ω-Dihydroxy-polyisobutylene (HO**–**PIB–OH)

The α,ω-dichloro-polyisobutylene (Cl–PIB–Cl) was obtained by the QLCCP of IB initiated by the *tert*-butyldicumyl chloride/TiCl_4_ initiating system in the presence of the TMEDA nucleophilic additive. Afterward, the Cl–PIB–Cl was subjected to three quantitative end-group modifications, as reported earlier.^61–64^ Briefly, the QLCCP of IB, producing the Cl–PIB–Cl, was end-quenched in situ by ATMS to obtain α,ω-diallyl-polyisobutylene (Al–PIB–Al). Subsequently, the purified Al-PIB-Al was regioselectively hydroborated and then oxidized to yield the desired α,ω-dihydroxy-polyisobutylene (HO–PIB–OH)—see Figure S1 in the Supporting Information (SI). The HO–PIB–OH was purified by first dissolution in *n*-hexane, passage of the solution through an alumina column, and precipitation of the effluent from the column in methanol. Finally, the precipitated polymer was dried under vacuum until constant weight at 40 °C.

#### Synthesis of α,ω-Dibenzaldehyde-polyisobutylene (Bz**–**PIB**–**Bz)

First, HO–PIB–OH of *M*_*n*_ = 10,000 g mol^–1^ (1 g, 0.1 mmol), 4-formylbenzoic acid (0.36 g, 2.4 mmol) and 4-dimethylaminopyridine (0.015 g, 0.12 mmol) were dissolved in absolute THF (dried over CaH_2_ and freshly distilled) in a round-bottomed flask. Afterward, *N,N′*-dicyclohexylcarbodiimide was dissolved separately in absolute THF, and the resulting solution was added dropwise into the flask containing the polymer reaction mixture, under stirring. The mixture was allowed to stir at room temperature for 3 days. Subsequently, the mixture was transferred into a benzoylated cellulose membrane and dialyzed against DMF for 3 days, with the external DMF being changed every day. Then, the mixture was dialyzed against benzene for 3 days with the external benzene being changed every day. Afterward, the dialyzed solution was first freeze-dried before it was dried under vacuum at 50 °C. The Bz–PIB–Bz was isolated as a transparent viscous liquid at a 85% yield (0.85 g, 0.085 mmol).

#### Synthesis of the Bz–PIB**–**Bz – tetraPEG–Hz Amphiphilic Polymer Conetworks

The three amphiphilic polymer conetworks (APCNs) were prepared by mixing the linear Bz–PIB–Bz with one of the three tetraPEG–Hz four-armed star homopolymers of molar mass 5000, 10,000, or 20,000 g mol^–1^, at their stoichiometric molar ratio, at a total polymer concentration equal to 7.5% w/v, and in the presence of glacial acetic acid as catalyst. As an example, the experimental procedure for the preparation of the APCN based on the tetraPEG–Hz of 20,000 g mol^–1^ and Bz–PIB–Bz is given in the following. First, Bz–PIB–Bz (0.022 g, 2.2 μmol) and tetraPEG–Hz of 20,000 g mol^–1^ (0.023 g, 1.12 μmol) were dissolved separately in benzene (300 μL). An alternative solvent would be toluene, which, however, is not readily freeze-driable as benzene. Then, glacial acetic acid (2 μL) was added into the Bz–PIB–Bz solution. Subsequently, the Bz–PIB–Bz and tetraPEG–Hz solutions were mixed, and the mixture was left at room temperature for 1 week before any further use. The above same procedure was followed using the two other tetraPEG stars, the tetraPEG–Hz of 5000 and 10,000 g mol^–1^ to prepare the two other APCN homologues.

### Methods

#### Proton Nuclear Magnetic Resonance (^1^H NMR) Spectroscopy

The purity, structure and end-functionality of all polymers and their derivatives were determined via ^1^H NMR spectroscopy in deuterated solvents, benzene-*d*_6_ or CDCl_3_, using either a Bruker 500 MHz Avance spectrometer or a Varian Inova 500 MHz spectrometer. ^1^H NMR spectroscopy was also used to follow the kinetics of the APCN formation reaction between Bz–PIB–Bz and tetraPEG–Hz. The details for the procedure followed to monitor the kinetics of the reaction between the tetraPEG–Hz of 20,000 g mol^–1^ and Bz–PIB–Bz are given below as an example. First, 0.022 g (2.2 μmol) of the Bz–PIB–Bz and 0.023 (1.12 μmol) g of the tetraPEG–Hz of 20,000 g mol^–1^ were dissolved separately in 300 μL of benzene-d_6_. Then, 2 μL of glacial acetic acid was added into the Bz–PIB–Bz solution. Subsequently, the Bz-PIB-Bz and tetraPEG–Hz solutions were mixed at room temperature, and the resulting reaction mixture was immediately transferred into an NMR tube. Afterward, the ^1^H NMR spectra were recorded at 0.25, 1, 3, 8, 21, 28, and 48 h after the transfer of the reaction mixture into the NMR tube. From those ^1^H NMR spectra, the conversion of the terminal benzaldehyde groups of Bz–PIB–Bz to acylhydrazone groups was calculated. The same procedure was also followed for the formation of the two other homologous APCNs based on the tetraPEG–Hz stars of 5000 and 10,000 g mol^–1^.

#### Gel Permeation Chromatography

The molar masses and molar mass distributions of the polymers were characterized by gel permeation chromatography (GPC) using a 1260 Infinity II Agilent liquid chromatograph equipped with an isocratic pump, a refractive index detector, and a single PLgel-5-μm mixed-D GPC column from Polymer Laboratories. The mobile phase was THF, delivered using the 1260 Infinity II isocratic pump at a flow rate of 1 mL min^–1^. Calibration was performed using near-monodisperse linear poly­(methyl methacrylate) molar mass (600–250,000 g mol^–1^) standards, whereas the molar masses and their dispersities were calculated using an OpenLAB CDS Workstation Software from Agilent.

#### Degrees of Swelling in DMF and Water

The degrees of swelling of the three APCNs in DMF and water were determined gravimetrically. For each measurement, five different samples were employed. Initially, the APCNs were transferred from the synthesis glass vials into larger glass vials containing 7 mL of DMF, so as to replace the synthesis solvent, benzene, with DMF. The supernatant DMF was refreshed every day, for 7 days, for the solvent exchange to be complete and the APCNs to be equilibrated in DMF. At this point, the DMF-swollen APCNs were weighed. Afterward, DMF was similarly exchanged for water until equilibration, and the water-swollen APCNs were weighed too. Finally, the APCNs were dried in a vacuum oven until constant weight, and the dried APCN masses were determined gravimetrically. The degrees of swelling in DMF and water were calculated by dividing the swollen by the dry conetwork mass. The degrees of swelling of selected samples (PIB–TetraPEG-20k) in toluene and *n*-hexane were also measured following a procedure similar to that described above.

#### Tensile Measurements

The three water-equilibrated APCNs were evaluated in terms of their tensile mechanical properties using an Instron 5944 mechanical testing apparatus from Instron, Norwood, MA, USA. Five to eight fresh samples were utilized for characterizing each of the three APCN samples, at a rate of extension equal to 30 mm min^–1^.

#### Small-Angle Neutron Scattering

The D22 instrument at the Institut Laue-Langevin—The European Neutron Source (ILL, Grenoble, France) was used for the small-angle neutron scattering (SANS) measurements (DOI: 10.5291/ILL-DATA.EASY-1417) at two sample-to-detector distances, 1.4 and 17.6 m, thereby covering a range of the magnitude of the wavevector, *q*, from 0.0025 to 0.6400 Å^–1^. The samples, placed in 2 mm thickness quartz cuvettes, were measured in D_2_O at 25 °C, in the bulk at 25 °C after thermal annealing overnight at 100 °C, and again in D_2_O (via rehydration after the thermal annealing) at 25 °C.

#### Dissipative Particle Dynamics (DPD) Simulations

DPD simulations^65^ were performed on three bulk APCNs based on ABA triblock copolymers end-linked at tetra-functional cross-links. The DPD reparametrization by Huang and Alexander-Katz^66^ was employed, where the density is increased from 3 to 5, and faithfully reproduces the experimental and theoretical morphology phase diagrams for linear diblock bulk melts. All three systems comprised the same midblock composed of 16 “red” beads (one bead may typically represent several monomer repeating units), and different end-blocks each composed of 2, 4, and 8 “blue” beads (see Figure 1d), thereby corresponding to “blue” volume fractions of 0.20, 0.33, and 0.50, close to the compositions in the three studied experimental APCNs. The above-simulated systems may also be viewed as consisting of four-armed star diblock copolymers, comprising 41, 49, and 65 beads (one extra “black” bead represents the star’s core, Figure 1d) end-linked at bifunctional linkers. In the simulations, each of the three APCN systems comprised 1728 of the corresponding four-armed star diblock copolymers, in a 12 × 12 × 12 arrangement. Thus, the three systems comprised 70,848, 84,672, and 112,320 beads. The χ*N*-incompatibility strength for the smallest system, also consisting of the shortest chains of 20 beads in each chain, was taken equal to 80,^38^ whereas the χ*N*-incompatibility strengths for the two other systems with longer chains of 24 and 32 beads were taken proportionately higher, equal to 96 and 128, respectively. To accelerate the very slow equilibration known to take place in cross-linked polymer systems,^38^ especially the two larger APCNs, both “solvent”-annealing and thermal-annealing were applied. In particular, the χ*N*-incompatibility strength was increased from 0 to the appropriate (final) value (“solvent”-annealing), e.g., 128, in steps of 10, within 24 million DPD steps, whereas this was followed by increasing the normalized temperature, *T*, from 1.0 up to 1.3 and then gradually lowering it back to 1.0 within another 10 million DPD steps.

**Figure 1:**
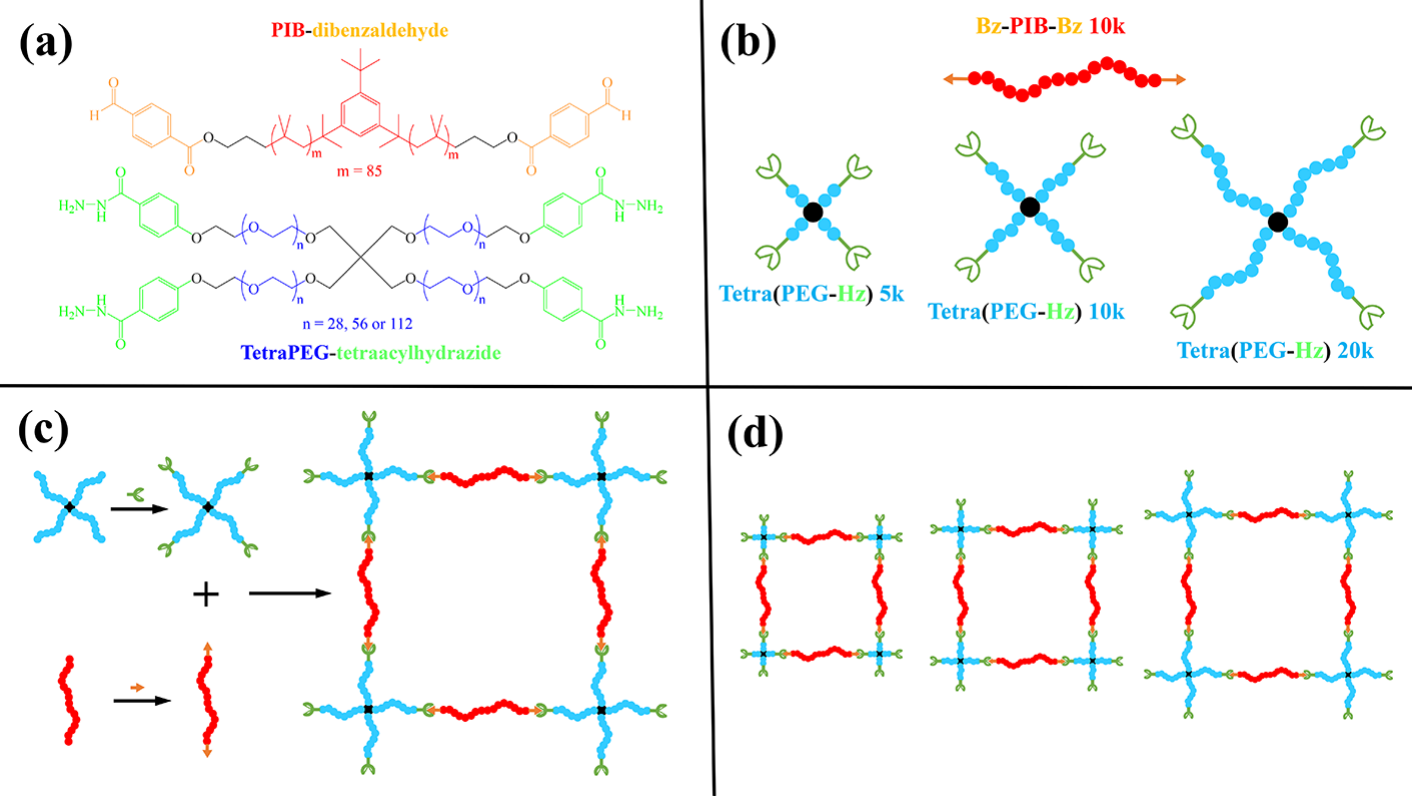
(a) Chemical structures and (b) diagrammatic representations (“cartoons”) of the end-functionalized polymeric building blocks, (c) cartoons illustrating the end-functionalization and interconnection of the polymeric building blocks to yield the APCN, and (d) cartoons of the homologous series of the three APCNs prepared and characterized in this study.

#### Molecular Thermodynamic Modeling

The prevailing morphology in the water-swollen APCNs was determined by the numerical minimization of the total Gibbs free energies for various possible morphologies. The prevailing morphology was identified as the one with the lowest total Gibbs free energy minimum. The lamellar, cylindrical and spherical morphologies, as well as the disordered state, thereby totaling four different polymer chain arrangements/states, were considered. All states were described by an elastic Gibbs free energy component and a mixing Gibbs free energy component. Regarding the latter component, it contained both entropic and enthalpic contributions for the disordered state, while it contained only an entropic contribution for the three ordered morphologies, in which the enthalpic contribution of the mixing component was replaced by the interfacial Gibbs free energy representing the residual contact between the two formed nanophases. All parameters employed for the calculations are listed in Table S2 in the SI, whereas the relevant equations^67–69^ were solved numerically using MATLAB^70^ on a laptop computer. The values of the minimized total Gibbs free energies for the four possible states of the three APCNs are displayed in Table S3 in the SI.

## Results and Discussion

### Chemistry and Architecture of Polymeric Building Blocks and Structure of APCNs

Figure 1 illustrates the chemical structures and diagrammatic representations (“cartoons”) of the end-functionalized PIB and PEG building blocks used for the preparation of the APCNs of this study, their interconnection through end-linking, and the three homologous APCNs thereby formed and characterized. The molar mass in kDa of each building block is indicated in part (b) of the figure. Whereas the hydrophobic PIB is used in linear form, the hydrophilic PEG is used in the form of a four-armed star homopolymer (“tetraPEG star”) also serving as cross-linker. Although the two types of building blocks are both homopolymers, their combination to yield the network results in the formation of PEG–PIB–PEG triblock copolymers constituting the APCN elastic chains (chains between cross-links). Details of the end-functionalizations of the PIB and the tetraPEG stars, and the preparation of PIB from the isobutylene monomer via quasiliving carbocationic polymerization (QLCCP)^61,63,64^ can be found in the Experimental Section and the Supporting Information (SI, Figures S1–S3 and Table S1). The following section outlines the preparation of the APCNs from the combination of the end-functionalized building blocks, and a problem that appeared during that preparation and had to be solved to secure conetwork formation and homogeneity.

### APCN Formation

Figure 2 shows the (partial) chemical structure of the APCN obtained by mixing together the two polymeric components, the hydrophobic Bz-PIB-Bz and the hydrophilic tetraPEG–Hz, each possessing terminal functional groups that can react with those of the other. In order to obtain the APCNs, concentrated (∼15% w/v) solutions of each polymer in benzene (toluene could be used instead), a common solvent, containing 1% w/v acetic acid to catalyze the acylhydrazone^71–73^ formation reaction, were prepared and mixed together at the stoichiometric ratio of the polymer end-groups, in order to induce gel formation. Surprisingly, no gel was formed! Instead, two liquid phases (not very viscous) were formed, apparently due to polymer solution demixing (macrophase separation). Analysis of each liquid phase using ^1^H NMR spectroscopy indicated that the top phase consisted exclusively of a PIB solution in benzene, whereas the bottom phase contained a tetraPEG-rich benzene solution of a PIB– tetraPEG mixture. By preparing gradually more dilute polymer solutions and mixing them, it was established that polymer solutions of concentrations lower than 10% w/v were fully miscible. Thus, it was decided to form the gels at a total polymer mass concentration of 7.5% w/v throughout this study. This lower-than-usual polymer concentration would slow down the kinetics of cross-link formation, and, consequently, gelation (also see following section). The presently determined critical polymer concentration for demixing of 8–10% w/v is 3–4 times lower than that determined for a previously investigated PIB–PEG APCN system whose critical demixing polymer concentration was ≥30%.^74^ However, the molar masses of the polymeric components in that previous APCN system (whose hydrophobic and hydrophilic components were not arranged in an alternating fashion because of the isocyanate linking chemistry employed) were low, about 1 order of magnitude lower than those in the present one, thereby shifting macrophase separation to higher polymer concentrations.

**Figure 2:**
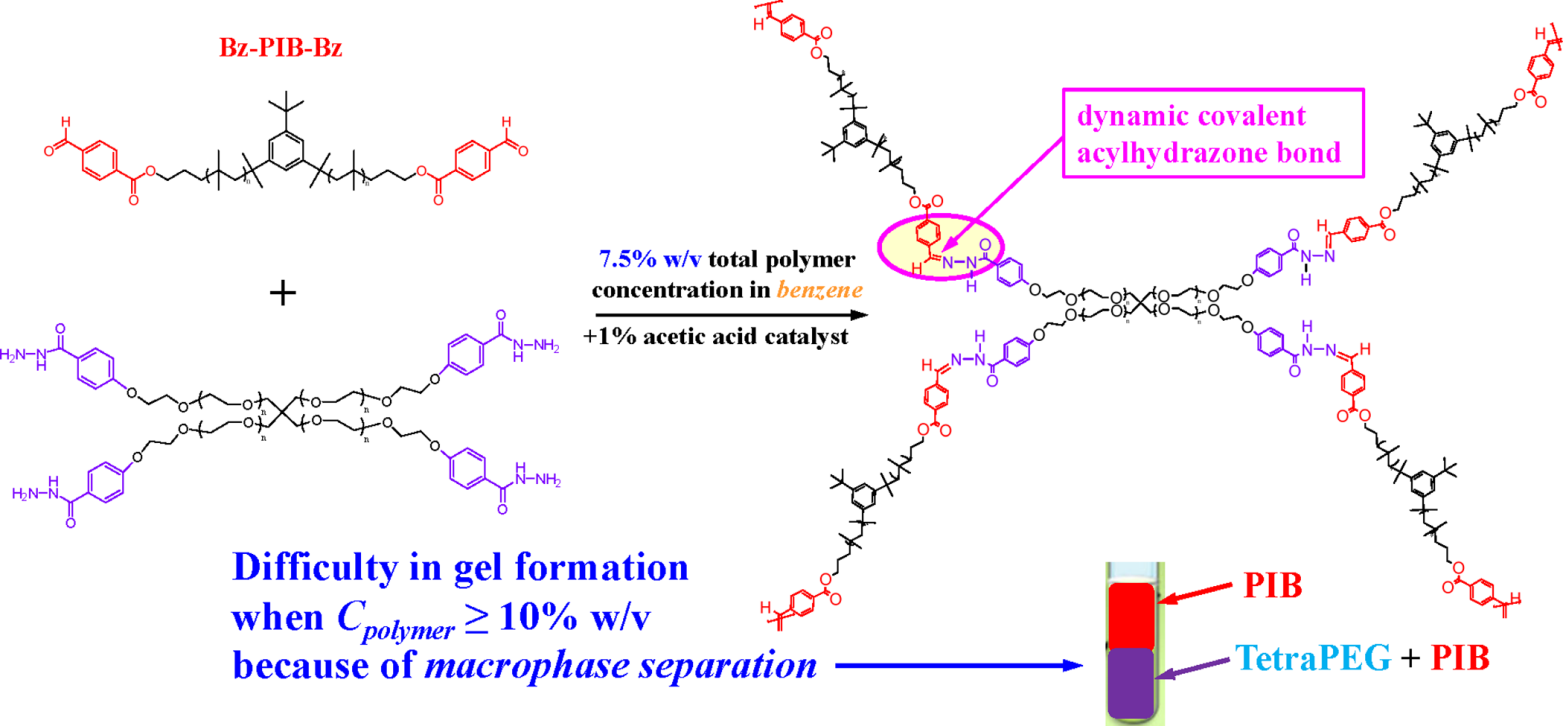
Mixing of the two polymeric components to form APCNs.

In Figure 2, one may see that the two types of end-groups, benzaldehyde and acylhydrazide, react with each other to form the cross-link, which is an acylhydrazone group.^71,72^ This group contains, among others, a carbon–nitrogen double bond, similar to a Schiff base, but much more stable than a Schiff base, yet reversible/exchangeable under acidic conditions.^73^ However, within the scope of this initial study, no acylhydrazone cross-link reversibility investigations have been performed.

### Kinetics of the Gel Formation Reaction for the Bz**–**PIB**–**Bz–TetraPEG-10k Mixture in Benzene-*d*_6_ Using Simple Solution ^1^H NMR Spectroscopy

Whereas it was difficult to follow, using ^1^H NMR spectroscopy, cross-link formation in our previous work on polymethacrylate-based APCNs where ethylene glycol dimethacrylate (EGDMA) or other divinyl molecules were used as cross-linkers,^19–21,39,40,50,52^ this is not the case in our recently developed APCN systems bearing acylhydrazone cross-links. In particular, with these systems, it is now possible to follow, using simple solution ^1^H NMR spectroscopy (no need to employ solid-state NMR spectroscopy using magic-angle spinning which require highly specialized and expensive spectroscopy equipment) the temporal evolution of the acylhydrazone cross-link formation from the disappearance of the aldehydic proton at ∼10 ppm.^49,59^ Figure 3 below presents the relevant consecutive solution ^1^H NMR spectra of the gel-forming Bz–PIB–Bz–tetraPEG-10k reaction mixture in benzene-*d*_6_ for a period of 2 days during which cross-link formation rises from 9% at 15 min to 83% in 48 h. The conversions to acylhydrazone cross-links for all seven times are also indicated in the respective ^1^H NMR spectrum.

**Figure 3:**
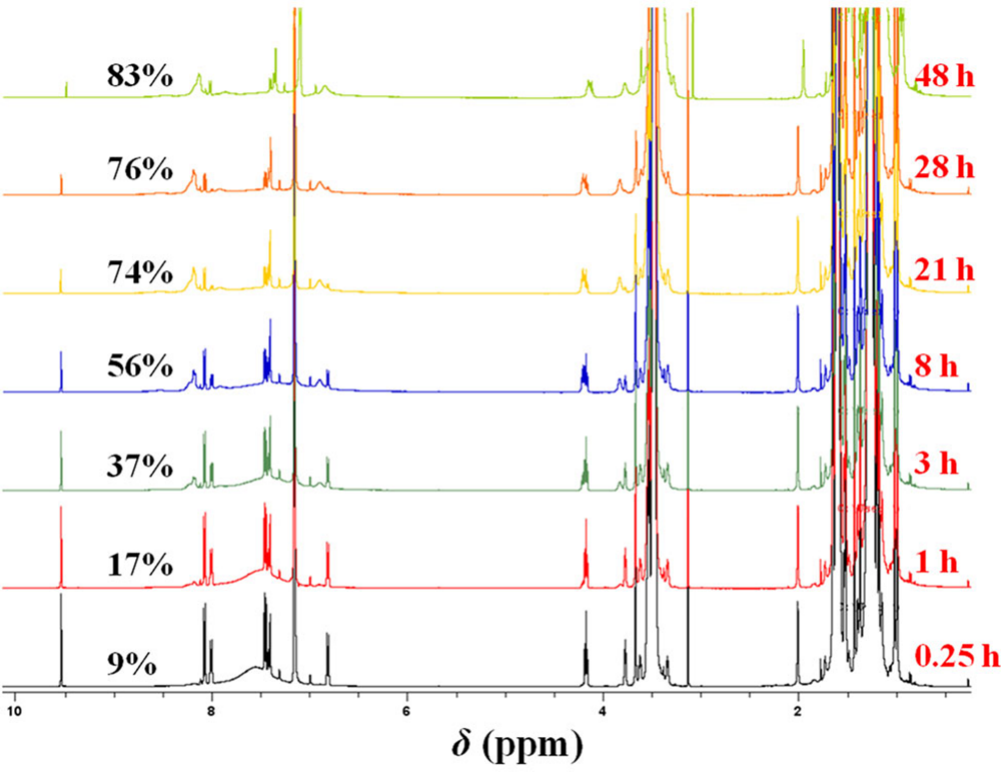
Sequential ^1^H NMR spectra of PIB–TetraPEG-10k in benzene-*d*_6_ leading to gel formation.

The time dependence of the conversion to acylhydrazone cross-links for this gel sample (Bz-PIB-Bz–tetraPEG10k) is plotted in the next figure, Figure 4, together with the corresponding data concerning the other two APCNs based on tetraPEG star cross-linkers of 5 and 20 kDa.

**Figure 4:**
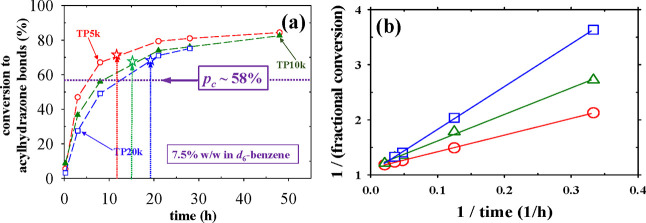
(a) Temporal evolution of the percentage conversion to acylhydrazone cross-links in the three homologous APCNs determined using ^1^H NMR spectroscopy in *d*_6_-benzene. (b) Second-order plot.

### Second-Order Rate Plots and Gel Points

Figure 4a presents the conversion to the acylhydrazone cross-link group for all three APCN samples vs time, from the beginning of the reaction (at 15 min) up to 48 h, with all reactions having been performed at a constant total polymer mass concentration of 7.5% w/v. The temporal evolution of the conversion for all three gels has the characteristic concave-down shape, typical for second-order reaction kinetics, with faster increase in the beginning, and leveling off in later stages, exceeding 80% in 48 h. The APCN cross-linked with the smallest tetraPEG star of 5 kDa reacts the fastest, followed by that cross-linked with the tetraPEG star of 10 kDa, and the slowest being the APCN cross-linked with the tetraPEG star of 20 kDa. This trend can be explained by the fact that, at a constant **total polymer** *mass* **concentration of 7.5% w/v**, the highest molar end-group concentration is displayed by the tetraPEG star of the lowest molar mass of 5 kDa followed by that of 10 kDa and then by that of 20 kDa. The critical conversion to acylhydrazone cross-links for gel formation (determined by inversion of the NMR tube) is indicated by the hollow star symbols placed on the relevant kinetic curves. For all three APCNs, this critical conversion is about 70%, approximately 12% higher than the critical conversion of 58% predicted by the Flory–Stockmayer theory.^17^ This discrepancy may be attributed to loop formation in the experimental system; these formed loops are elastically inactive, contributing to the conversion to acylhydrazone bonds, but not to gel formation.

The data in the above figure are linearized, by plotting the inverse of the fractional conversion to acylhydrazone groups vs the inverse of the reaction time (Figure 4b), appropriate for second-order kinetics with the reacting species being at their stoichiometric ratio. The slopes in the plots are equal to 1/(*k* × [A]_0_), with *k* being the second-order reaction rate constant, and [*A*]_0_ being the initial molar concentration of the reactive end-groups, i.e., the benzaldehyde terminal groups whose (initial) concentration is equal to that of the acylhydrazide ones. Because of data inversion, the highest slope is displayed by the 20 kDa tetraPEG star-containing system, followed by those of the 10 and 5 kDa.

The values of the calculated slopes are listed in the following table, Table 1, together with the initial benzaldehyde end-group molar concentrations, as well as the determined second-order reaction rate constants. The values of the second-order reaction rate constants, together with their standard deviations, are plotted against the values of the molar masses of the tetraPEG stars in Figure S4 in the SI. The values of the second-order reaction rate constants, ***k***, for the three APCN systems are similar to each other, but not identical (this would have been ideal), differing beyond statistical error, also listed in the table, and calculated as the error propagated from the standard deviation of the slope. The second-order reaction rate constants decrease with the tetraPEG star cross-linker molar mass, possibly attributable to the larger steric hindrances in the presence of the longer arms in the systems with the larger stars.

**Table 1:** Second-Order Reaction Rate Constants for the Acylhydrazone Group Formation in the APCNs

no.	tetraPEG molar mass (g mol^–1^)	slope (h)	[*A*]_0_ (mM)	*k* (M^–1^ h^–1^)
**1**	5,000	3.016 ± 0.023	14.29	23.21 ± 0.18
**2**	10,000	4.812 ± 0.130	11.54	18.01 ± 0.49
**3**	20,000	7.770 ± 0.062	8.333	15.44 ± 0.12

### Degrees of Swelling of the PIB-TetraPEG APCN Gels in Water and DMF

The most basic thermodynamic property of polymer gels is their equilibrium swelling behavior in various environments. The network swelling degrees in different solvents is easy to determine experimentally, most usually gravimetrically. Yet, these degree of swelling values highly influence other APCN properties, including the mechanical and self-organization properties. Figure 5 presents the degrees of swelling of the PIB–TetraPEG gels in water and DMF, after having been exchanged originally from benzene (common solvent for PIB and PEG) and then successively equilibrated into DMF and then into water. Both DMF and water are good solvents for PEG and poor solvents for PIB. Moreover, DMF is miscible both with benzene and water (and is, consequently, an excellent intermediate solvent), and it also facilitates the unpeeling of the gels from the preparation glass vials. The figure shows that the degrees of swelling in the two solvents are very similar, statistically identical given the standard deviations also illustrated in the figure, increasing linearly from about 3 up to about 8 with the molar mass of the constituting tetraPEG stars. The similar swelling values in the two solvents suggest that the two solvents exhibit approximately the same affinities toward each of the two APCN components, PEG and PIB. The slightly higher swelling in DMF as compared to water is consistent with the notion that water is a poorer solvent for PIB than the organic (but polar) DMF. The increase of the degree of swelling in both water and DMF with the tetraPEG star molar mass may be attributed to two factors. The first factor is that, as the tetraPEG star molar mass increases, the content in PEG (for which both water and DMF are selective solvents), relative to PIB, also increases in these stoichiometrically prepared conetworks. The second factor is that a higher molar mass of the tetraPEG stars, also having the role of cross-linker, leads to longer elastic chains (PEG–PIB–PEG triblock copolymers), and, consequently, a lower cross-linking density, thereby resulting in a higher swelling of the present conetworks in both water and DMF.

**Figure 5:**
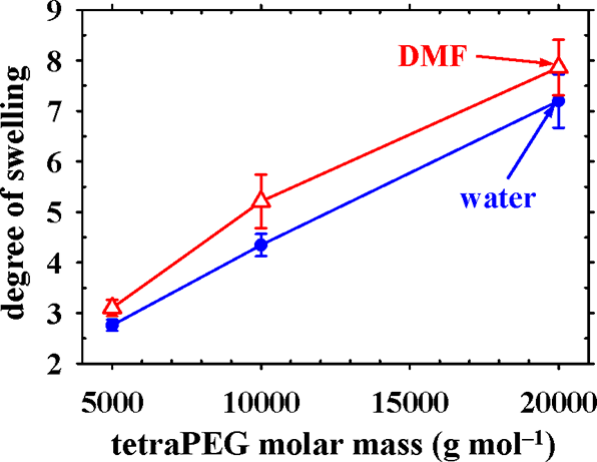
Dependence of the degrees of swelling of the PIB–tetraPEG APCNs in water and DMF on the molar mass of the constituting tetraPEG stars.

Further swelling measurements were performed on the PIB–TetraPEG-20k APCN. The degree of swelling of this sample in toluene was determined to be 13.21 ± 0.13. This relatively high swelling value is due to the fact that toluene is a nonselective solvent, dissolving both PIB and PEG. On the other hand, the degree of swelling of the same sample in *n*-hexane, which is selective for PIB, was expectedly found to be lower, at 1.84 ± 0.15. Finally, the swelling degree of PIB–TetraPEG-20k in water, after the sample had been thermally annealed under vacuum overnight at 100 °C, was calculated to be 2.73 ± 0.03, much lower than 7.20 ± 0.53 determined without annealing. This reduction in aqueous swelling upon thermal processing might be attributed to the melting of a small amount of PEG crystallites present in the unprocessed sample. These crystallites may lead to the formation of a less compact structure, resulting in higher aqueous swelling, and also destroying long-range ordering in the structure of this sample (see following section on Self-assembly).

### Tensile Properties of the PIB**–**TetraPEG APCN Gels in H_2_O

A key property of interest of the developed materials is their mechanical response to deformation. Figure 6 displays the tensile measurements for the mechanical characterization of the three APCNs of this study equilibrated in water, after being transferred successively from benzene into DMF and then into water. For each different APCN, several measurements were performed on several (5–8) fresh samples, so as to secure good statistics, because hydrogels, even well-defined ones, are known not to be very reproducible when it comes to their mechanical characterization, especially in tension. Yet, the tensile stress–strain curves for each of the three APCNs presented in Figure 6 are reasonably reproducible, given their hydrogel nature.

**Figure 6:**
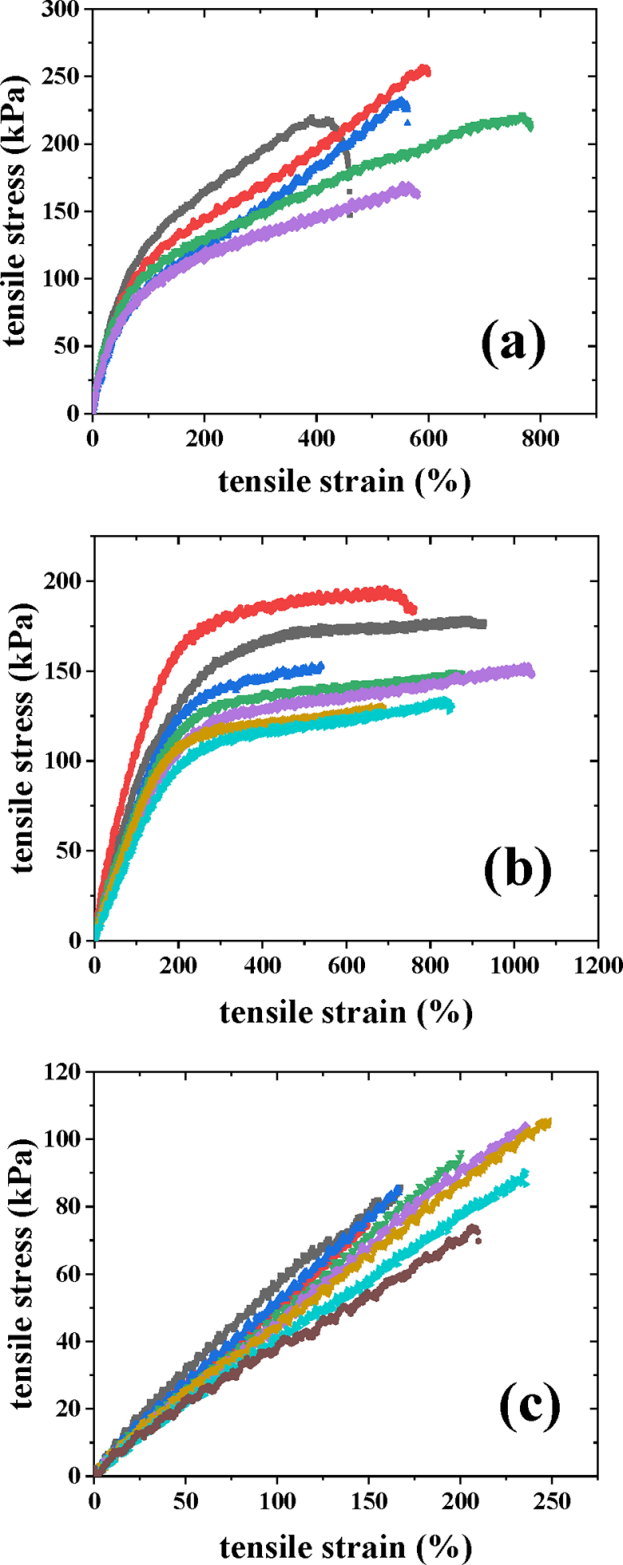
Tensile stress–strain curves (all repetitions) for the three PIB–TetraPEG APCN gels in water at room temperature. (a) PIB–TetraPEG-5k, (b) PIB–TetraPEG-10k, and (c) PIB–TetraPEG-20k. Sample dimensions were 10 mm (length between clamps) × 4.0 mm (width) × 1.6 mm (thickness), whereas the elongation rate was at 300% min^–1^.

Surprisingly, the tensile stress–strain curves for the three APCN samples display some fundamental differences from each other, especially for strains higher than 100–200% up to which all three conetworks exhibit a linear response. In particular, the TetraPEG-5k-based APCN exhibits a yielding-like behavior after the linear region, the TetraPEG-10k-based APCN presents a unique plateau similar to the one found in double-network gels^75^ after its linear region, whereas the TetraPEG-20k-based APCN appears to have only a linear region. These differences may be explained by considering the “loose-structure” vs the ability to form reversible physical linkages (“fuse-links”) in each APCN.^43^ The “loosest structure” is exhibited by the TetraPEG-20k-based APCN because of its longest elastic chains and highest aqueous swelling degree, followed by those of the 10k and 5k counterparts, in this order. On the other hand, the ability for “fuse-link” formation follows the reverse order, with the TetraPEG-5k-based APCN, which is the most hydrophobic, presenting the highest capability to form reversible hydrophobic linkages, and the less hydrophobic 10k and 20k counterparts occupying the second the third positions, respectively.

The TetraPEG-5k-based APCN is the one with the highest capability to form “fuse-links” because of its very high hydrophobic PIB content of about 80% (the PEG volume fraction is only about 20%). In principle, the physical separation among segregated PIB hydrophobic chains, upon tensile deformation, would impart a very high extensibility and toughness to this sample. However, the very dense structure, arising from the short elastic chains and the low aqueous swelling degree, does not allow high extensions, leading to the yield-like response after the linear region and failure at relatively high stress values and fair strain values. On the other extreme, the TetraPEG-20k-based APCN is the one with the “loosest structure,” due to its long elastic chains and high aqueous swelling degree. This very “loose structure” was expected to result in high extensibility. However, this extensibility is restricted within the linear region, reaching the fairly high strain at break values of ∼200%. The restriction is believed to be arising from the limited capability of this sample to form “fuse-links”. Finally, the TetraPEG-10k-based APCN is the one possessing the best mix of the two molecular properties: high enough capability to form “fuse-links” and sufficiently “loose structure”. Thus, after the linear region ending at a strain of 200%, this APCN can be extended for another ∼700% for some samples, reaching strains at break higher than 800%. On the molecular level, the long enough chains of the TetraPEG-10k-based APCN do not prohibit high extension, during which the self-assembled PIB hydrophobic components reversibly get separated and recombine, thereby serving as sacrificial bonds (note the striking similarity of the stress–strain curve of this sample to that of DN gels^75^) protecting this conetwork from early failure.

From these tensile stress–strain curves, the averages of four mechanical properties were determined: the tensile Young’s modulus, *E*_exp_, the tensile stress at break, σ_max_, the tensile strain at break, ε_max_, and the tensile toughness. These averages, together with their standard deviations, are listed in the following table, Table 2, and are plotted against the tetraPEG star molar masses in Figure S5 in the SI. The table also displays the equilibrium degree of swelling of the APCNs in water, *DS* (also presented and discussed in the previous section), and the theoretical Young’s modulus, *E*_theory_. The latter was simplistically calculated, assuming no nanophase separation takes place in water (this assumption will be examined later), for the three APCNs using the phantom network model and assuming conetwork incompressibility (Poisson’s ratio taken equal to 0.5), according to eq 1 below:^17^

$$E_{\mathrm{theory}}=3\times (1-2/f)\times \nu \times R\times T$$ 1

where *f* is the junction functionality, being equal to 4 in the present conetwork system because of the tetra-functional cross-links, ν is the molar concentration of the elastic chains in the conetworks calculated from the equilibrium swelling degree in water, *DS*, and the molar mass of the elastic chains, *MM*_elastic‑chain_, also listed in the table, with ν = 1/(*DS* × *MM*_elastic‑chain_), *R* is the universal gas constant, and *T* is the absolute temperature (=298 K).

**Table 2:** Tensile Mechanical Properties of the PIB-TetraPEG APCNs Equilibrated in Water

sample number	1	2	3
TetraPEG star molar mass (g mol^–1^)	5,000	10,000	20,000
Young’s modulus, *E*_exp_ (kPa)	220 ± 30	91 ± 19	52 ± 9
stress at break, σ_max_ (kPa)	217 ± 34	130 ± 34	85 ± 19
strain at break, ε_max_ (%)	470 ± 180	810 ± 160	200 ± 30
toughness (MJ m^–3^)	90 ± 22	106 ± 28	10 ± 3
degree of swelling in water, *DS*	2.76 ± 0.11	4.35 ± 0.22	7.20 ± 0.53
elastic chain molar mass (g mol^–1^)	12,500	15,000	20,000
ν (mM)	34.5	17.8	7.71
theoretical Young’s modulus, *E*_theory_ (kPa)	128.2	66.2	28.7
doubled theor. Young’s modulus, *E*_th‑cor_ (kPa)	256.4	132.4	57.4

The experimentally determined tensile Young’s modulus of the water-equilibrated APCNs decreases as the tetraPEG star cross-linker molar mass increases because of the increase in the aqueous swelling degree and the reduction in cross-linking density. The theoretical Young’s modulus calculated using the phantom network model^17^ presents the same reduction as the tetraPEG star molar mass goes up, for the same reasons given for the experimentally determined tensile Young’s modulus values. However, the values of the experimentally determined values are 40–80% higher than the theoretically calculated ones, due to the fact that the APCN self-organization in water was not taken into account in the calculation. When the “micellization” of the constituting PEG–PIB–PEG triblock copolymer chains is recognized, one may consider that the number of elastic chains doubles because, with the aggregation of the PIB central blocks, each PEG–PIB–PEG triblock elastic chain yields two PEG homopolymer elastic chains. This doubling in **ν** would lead to the doubling in the theoretically calculated Young’s modulus (last row in Table 2), whose values are satisfactorily close to the experimentally determined Young’s modulus values.^54^

The tensile stress at break, σ_max_, of the water-equilibrated APCNs decreases from 217 down to 85 kPa as the tetraPEG star molar mass increases from 5 to 20 kDa. This can be attributed to the higher cross-linking density and the lower aqueous swelling degrees for the APCN samples based on the smaller stars. Regarding the tensile strain at break, ε_max_, of the water-equilibrated APCNs, this was expected to display the opposite trend relative to the stress at break, i.e., ε_max_ was expected to monotonically increase as the tetraPEG star size increased. Instead, however, ε_max_ reproducibly exhibited a maximum for the APCN sample with the tetraPEG star of intermediate molar mass, 10 kDa. This maximum in tensile strain at break for the APCN based on the tetraPEG star of the intermediate molar mass may be attributed to the optimal molecular constitution of this particular APCN, possessing a high enough hydrophobic content so as to exhibit efficient “fuse-link” behavior and long enough elastic chains to be able to display sufficient extensibility (see discussion in a previous paragraph of this section). As a result, the extensibility of this APCN is remarkable, with one of its samples reaching the record value of ε_max_ being equal to 1100%, despite the high equilibrium water content in this APCN, reaching 77%! The optimal composition of this APCN is also the reason why it presents the highest toughness of 106 MJ m^–3^, much higher than that of the 20 kDa tetraPEG star-based counterpart, and slightly higher than that of the 5 kDa tetraPEG star-based counterpart.

Extra tensile experiments were performed on the PIB–TetraPEG-20k APCN. These experiments aimed at investigating the reversibility of the elongation, and exploring the tensile behavior after thermal annealing. Regarding the former type of experiment, the sample was simply stretched to 100% strain, and was, subsequently, released and allowed to return to its original position. The sample returned to almost its original length, displaying only a 3.55% residual extra length, representing only a small irreversibility. This behavior is highly elastic (by 96.45%), which may also originate from the “fuse-link” action of the rubbery PIB segments. The small permanent deformation may originate from viscous effects, and not from the dynamic covalent cross-links, which, in the absence of acid (acetic acid was thoroughly removed through the multiple washings performed) are frozen, exhibiting lifetimes in the order of days,^54^ and essentially behaving as conventional covalent bonds.

Four thermally annealed samples of the PIB–TetraPEG-20k APCN were also characterized in tension using the Instron mechanical tester, and the recorded tensile stress–strain curves are illustrated in Figure S6 in the SI. The shape of the stress–strain curves of these annealed samples was clearly concave down, substantially different from those of the original, non-annealed, samples shown in Figure 6c which were almost linear. The tensile strain at break of the annealed samples [=(190 ± 50)%] was essentially the same as that of the nonannealed ones [=(200 ± 30)%], indicating the same chain configuration during fracture for the two types of samples. However, upon annealing, the Young’s modulus and the stress at break increased. The Young’s modulus of the annealed samples was (420 ± 120) kPa, higher than the Young’s modulus value before annealing of (52 ± 9) kPa, and consistent with the reduction of the aqueous swelling degree upon annealing. Similarly, the stress at break in the annealed state was also higher, displaying a value of (380 ± 120) kPa, as compared to (85 ± 19) kPa which is the value before annealing.

### Self-Assembling Properties of the PIB**–**TetraPEG APCNs in D_2_O and in Bulk

The most characteristic property of APCNs is their ability to phase separate on the nanoscale when transferred into a selective solvent such as water. The design and synthesis of near-perfect APCNs aims at rendering this nanophase separation with long-range ordering, i.e., to have a regular arrangement of the hydrophobic and hydrophilic nanophases. Small-angle neutron scattering (SANS) in deuterated water, D_2_O, is a most appropriate method to detect APCN nanophase separation and its regularity, from the appearance of a main scattering (correlation) peak and higher-order peaks, respectively. Thus, SANS was performed on the three APCNs equilibrated in D_2_O, and the recorded SANS profiles are plotted in Figure 7a below. In these SANS profiles, the intensity of scattered radiation (neutrons) is plotted against the magnitude of the scattering wave vector, *q*.

**Figure 7:**
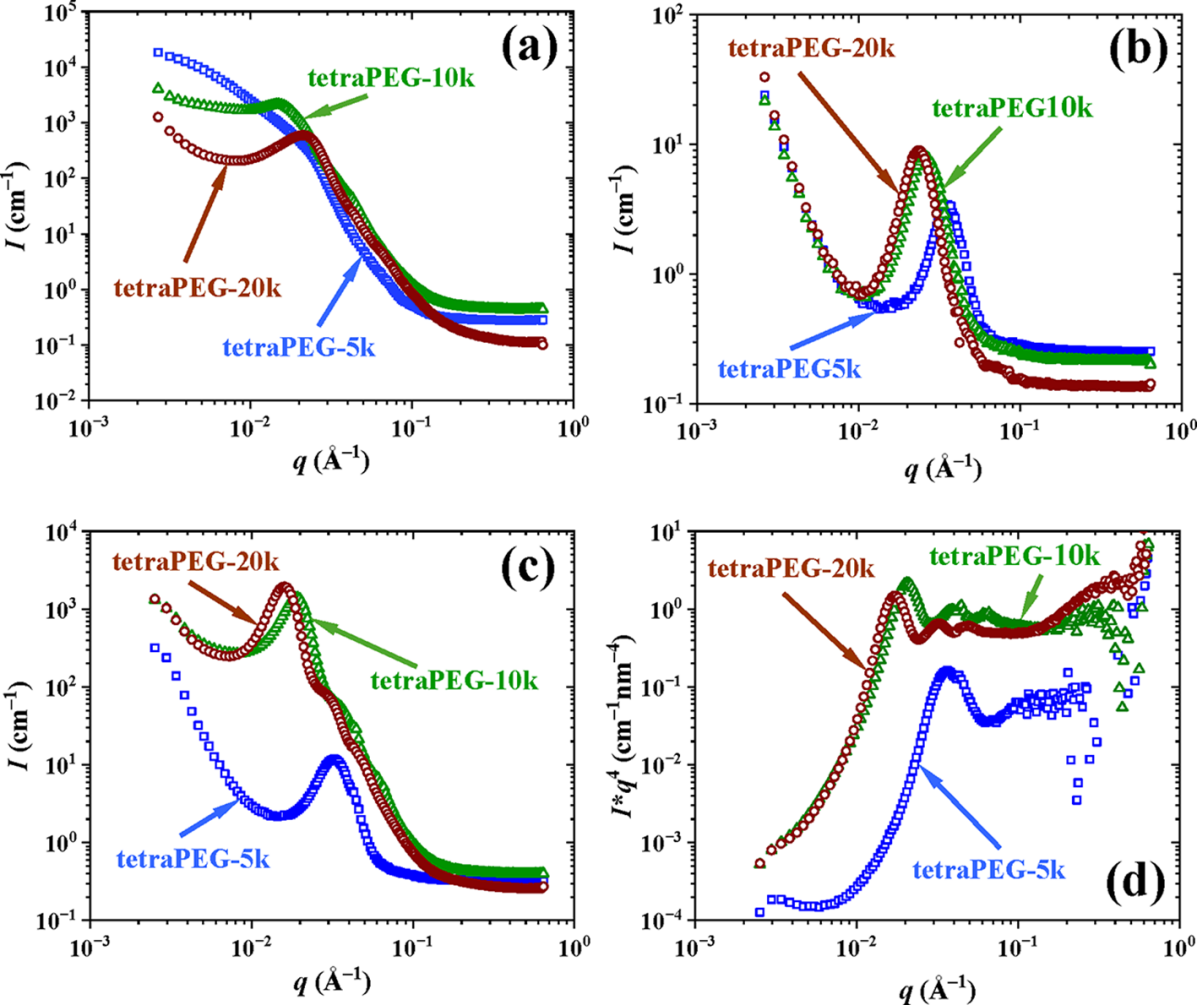
SANS profiles of the non-annealed and annealed PIB–TetraPEG APCNs in bulk and D_2_O. (a) Non-annealed samples in D_2_O, (b) annealed samples in the bulk, (c) annealed samples in D_2_O, and (d) Porod plot of the SANS profiles of the annealed samples in D_2_O shown in part (c).

The APCN based on the smallest tetraPEG star does not present a correlation peak but only a shoulder, indicating absence, or low quality, of nanophase separation. In contrast, the two APCNs based on the larger tetraPEG stars exhibit clear, albeit broad, correlation peaks, suggesting nanophase separation but without long-range ordering. From the position of the peaks, *q*_max_, one may calculate the spacings ***d***, between the scattering centers, as *d* = 2π/*q*_max_, which come out to be 42 and 30 nm for the APCNs based on the 10 and 20 kDa tetraPEG stars, respectively (see Table 3). The relative values of these spacings seem to contradict the relative sizes of the two tetraPEG star cross-linkers, and also the measured aqueous degrees of swelling of the corresponding APCNs. Thus, it is possible that the APCNs characterized using SANS in D_2_O were not yet brought into their equilibrium configuration, most likely due to some small crystallinity in the PEG phase (although this crystallinity usually vanishes upon sample hydration), which makes the sample porous and less compact, absorbing more water, and exhibiting only short-range order. In order to test this hypothesis and help bring the APCNs in their equilibrium state, it was decided to thermally anneal the samples above the melting temperature of PEG of 67 °C, rehydrate them in D_2_O, and reexamine them using SANS. Given the fact that PIB and PEG have significantly different scattering length densities,^62^ it was decided to also reexamine the samples using SANS in the bulk right after their thermal annealing and before their rehydration.

**Table 3:** Peak Spacing from SANS for the PIB–TetraPEG APCNs in D_2_O before and after Thermal Annealing, and in the Bulk after the Thermal Annealing, as well as Some Characteristic Distances Calculated from the Molecular Sizes of the Samples

sample number	1	2	3
TetraPEG star molar mass (g mol^–1^)	5,000	10,000	20,000
in D_2_O before annealing (nm)	---	42	30
in bulk after annealing, *d*_bulk_ (nm)	17	24	26
in D_2_O after annealing, *d*_D2O_ (nm)	21	35	40
elastic chain contour length (nm)	55	74	111
elastic chain coiled diameter in bulk, *coil*_bulk_ (nm)	3.26	3.47	3.82
elastic chain coiled diameter in D_2_O, *coil*_D2O_ (nm)	4.57	5.66	7.37

Thus, Figure 7b depicts the SANS profiles of the three APCNs in the bulk, after vacuum-drying and thermally annealing at 100 °C overnight in a vacuum oven, whereas part (c) of the same figure illustrates the corresponding SANS profiles in D_2_O by rehydration (via D_2_O addition) after being dried and thermally annealed. Finally, part (d) of the figure replots the data in part (c) but in Porod form, where the scattered neutron intensity in the *y*-axis is multiplied by the fourth power of *q*, *q* ^4^ *.* The SANS profiles of the three thermally annealed APCNs in the bulk in Figure 7b now all present correlation peaks, even the APCN based on the smallest tetraPEG star. Furthermore, the position of the correlation peaks, *q*_max_, of the three APCNs are in the correct order, corresponding to domain spacings of 17, 24, and 26 nm (Table 3 and Figure S7 in the SI), and increasing with the tetraPEG star cross-linker size, as expected. Thus, the peak positions of the APCNs based on the two larger tetraPEG star cross-linkers now appear to be in the right relative positions. It is noteworthy that the scattering signal in the SANS profiles for the APCNs presented in this part of the figure, part (b), is much weaker than that in part (a) which illustrates the SANS profiles in D_2_O. This is due to the lower contrast in the former case. Yet, the scattering contrast between bulk PIB and PEG is large enough to produce clear SANS profiles.

Upon the addition of D_2_O and rehydration of the APCNs, the SANS profiles acquire higher intensity, preserve the same correct peak order, but are shifted to lower *q*-values, with the correlation peaks corresponding to larger spacings, arising from the hydration and swelling of the PEG hydrophilic nanodomains and the shift of the segregated hydrophobic PIB nanodomains further apart from each other. These domain spacings are now 21, 35, and 40 nm (Table 3 and Figure S7), which must be compared to the previous spacings in the bulk of 17, 24, and 26 nm (again in Table 3 and Figure S7). Importantly, the SANS profiles of the APCNs based on the two larger tetraPEG star cross-linkers also present secondary peaks, suggestive of long-range ordering. To better visualize these weak peaks, the SANS profiles of these two APCNs are plotted in Porod form (part (d) of Figure 7), where the scattered intensity is accentuated by being multiplied by *q* ^4^. In the Porod plot presented in Figure 7d, the two secondary peaks in each sample now appear very clearly. Moreover, their relative positions with respect to the main peak, *q*_max_, is 2 × *q*_max_ and 3 × *q*_max_. This is consistent with a lamellar morphology in these two rehydrated samples. Note that the aqueous degree of swelling of the PIB–TetraPEG-20k APCN sample is reduced upon thermal annealing from a value of 7.2 down to 2.7 (see previous section on Degrees of Swelling), with the latter value being compatible with a lamellar structure.

### APCN Bulk Morphologies by Dissipative Particle Dynamic (DPD) Simulations

Thus, the annealed APCNs containing the two larger tetraPEG stars form **lamellae** in D_2_O, as this was deduced from the position of the higher-order peaks appearing in their SANS profiles. Because of the absence of higher-order peaks in the SANS profiles of the APCN samples in the bulk, it was not possible to decide on their morphologies. To shed light onto this issue, it was decided to perform coarse-grained simulations, dissipative particle dynamics (DPD)^65,66^ in particular, on these samples, by appropriately adjusting a code recently developed to study bulk APCNs on a diamond lattice.^38^ Whereas the diamond lattice arrangement (tetra-functional cross-links here too) was retained, the elastic chain architecture had to be modified from the AB diblock copolymer one used in our previously published simulations work^38^ to the ABA triblock one that applies here. The volume fractions of the “blue” end-blocks in the simulations were kept the same as in our experimental APCNs, at values of 0.20 (corresponding to the PIB–TetraPEG-5k APCN), 0.33 (corresponding to the PIB–TetraPEG-10k APCN) and 0.50 (corresponding to the PIB–TetraPEG-20k APCN). As in the experimental system too, the elastic chain length also increased with end-block volume fraction. The above were achieved by simulating the PIB red midblock as 16 beads (constant for all three APCN systems), and assigning to the PEG blue component 4 (2 in each end-block), 8 (4 in each end-block) and 16 (8 in each end-block) beads. After equilibrating the structures, with the aid of both “solvent” and thermal annealing, spheroids (“potatoes”), gyroids and lamellae were obtained for the APCNs with PEG volume fractions of 0.20, 0.33, and 0.50, respectively. Snapshots of these morphologies are illustrated in Figure 8. For the spheroidal morphology, where the “blue” nanophase is discontinuous, an aggregation number of 7 (presumably occupying the 6 vertices of a tetragonal bipyramid plus the central unit in that bipyramid) star block copolymers per spheroidal entity (28 diblock arms which are 28 half ABA triblock elastic chains) was estimated. This is a reasonable value for an aggregation number in the bulk, albeit being on the lower side. This lower value may be attributed to the presence of the cross-links which somewhat limit the aggregation number. This calculated aggregation number of 7 is reasonably close to those estimated experimentally by Nakagawa et al. at 17,^76^ and via simulations by Löser et al. also at 17,^77^ both on similar APCN systems comprising tetraPEG stars, but swollen in water; an aggregation number of 17 is suggestive of a star aggregation up to second-nearest neighbor,^76^ rather than first-nearest neighbor provided by our calculation. The discrepancy may be attributed to the greater driving force for self-organization in the presence of a selective solvent like water, as compared to the system at hand in the bulk. The presence of cross-links is also the reason why the blue spheroidal domains deviate from the spherical shape as the aggregating star elements are constrained by their cross-links which, in turn, create an asymmetrical force field.

**Figure 8:**
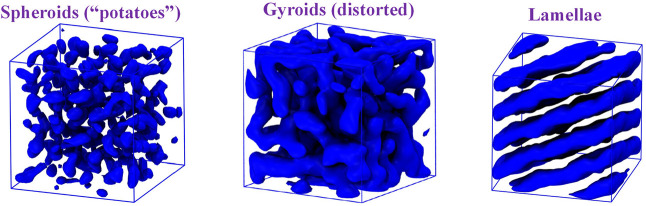
Snapshots of the bulk morphologies determined using DPD simulations for the three APCNs of this study. From left to right: PIB–TetraPEG-5k (PEG volume fraction = 0.20), PIB–TetraPEG-10k (PEG volume fraction = 0.33), and PIB–TetraPEG-20k (PEG volume fraction = 0.50).

### Lamellar Thicknesses

Although we would have liked to use our DPD simulation code^38^ to also determine the morphology of the tetraPEG-5k-based APCN in D_2_O, not exhibiting any higher-order peaks in its SANS profile, and to confirm the lamellar morphologies from the SANS results for the two other APCNs, we could not presently do this as our DPD methodology is not sufficiently developed for aqueous systems. Thus, we resorted to our molecular thermodynamic model^67–69^ specifically developed to determine the morphologies of APCNs in water. Following a numerical solution using MATLAB^70^ (see Methods in the Experimental Section/Methods and the SI), the model yielded that all three water-swollen APCNs equilibrate into lamellae. Knowing now that the morphology of all the APCNs is lamellar, we replotted in Figure 9 their SANS profiles in Kratky form (*I* × *q* ^2^ vs *q* ^2^). From the semilogarithmic plot, we first calculated the (negative) slope in the intermediate *q*-range, which is equal to (minus) the mean-square radius of gyration of the thickness, *R* *_c_* ^2^.^78^

**Figure 9:**
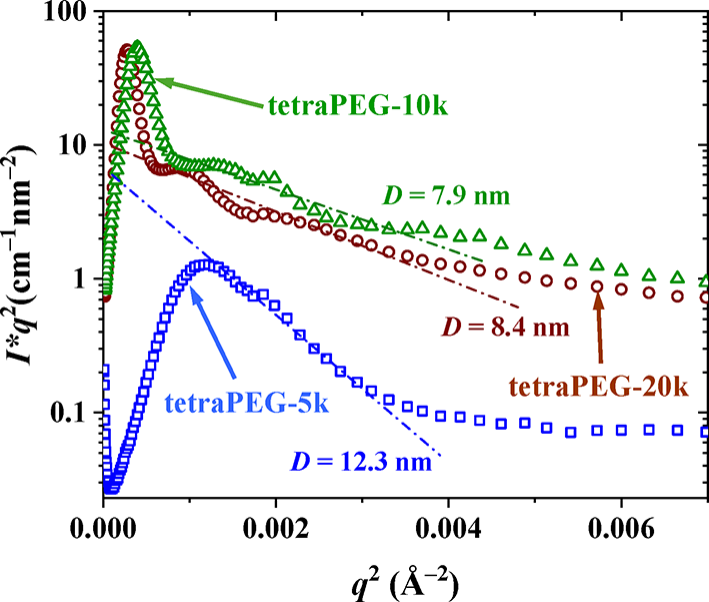
SANS profiles plotted in Kratky form for the calculation of the thickness of the hydrophobic lamellar layers for the three APCNs in D_2_O.

Subsequent multiplication of *R*_*c*_ times the square root of 12 yielded the thickness of the hydrophobic lamellar domains.^78^ Subtracting that from the domain spacing obtained from the primary peak positions, we could also estimate the thickness of the hydrophilic lamellar domains. All these lamellar domain sizes are listed in Table 4 (and plotted in Figure S8 in the SI), which also lists the relevant sizes corresponding to the molecular dimensions of the hydrophilic and hydrophobic segments.

**Table 4:** Thicknesses of the Hydrophobic and Hydrophilic Lamellar Layers, and Relevant Upper and Lower Limits of These Thicknesses, for the Three APCNs in D_2_O

no.	*MM*^a^ (kDa)	*d*^b^ (nm)	*D*^c^ (nm)	(*d*–*D*)^d^ (nm)	*D*_min_^e^ (nm)	*D*_max_^f^ (nm)	(*d*–*D*)_min_ ^g^ (nm)	(*d*–*D*)_max_ ^h^ (nm)
1	5	21	12.3	8.7	3.02	36.3	2.41	18.8
2	10	35	7.9	27.1	3.02	36.3	3.04	37.5
3	20	40	8.4	31.6	3.02	36.3	3.83	75.0

a *MM*: molar mass of tetraPEG star.

b *d*: dual layer spacing estimated from the position of the primary peak at *q*_max_, as *d* = 2π/*q*_max_.

c *D*: thickness of the PIB hydrophobic layer estimated from the slope in the Kratky plots.

d (*d*–*D*): thickness of the PEG hydrophilic layer estimated by subtraction of *D* from *d*.

e *D*_min_: diameter of an anhydrous (bulk) PIB sphere.

f *D*_max_: contour length of the PIB linear chain.

g (*d*–*D*)_min_: diameter of an anhydrous (bulk) tetraPEG star.

h (*d*–*D*)_max_: contour length corresponding to a linear chain consisting of two tetraPEG star arms.

The table shows that the thicknesses of the hydrophobic PIB layers, *D*, are not the same in the three APCNs, despite the same PIB segment as the middle block of the PEG–PIB–PEG triblock copolymers elastic chains. In particular, *D* slightly decreases with the tetraPEG star size, consistent with a less intense stretching of the hydrophobic layer in the more swollen hydrogels (where aqueous swelling mainly leads to the stretching of the PEG chains). However, in all three cases, the *D* values always expectedly remain intermediate between their theoretical minimum and maximum values, *D*_min_ and *D*_max_, also listed in Table 4 (and plotted in Figure S8), respectively estimated as the diameter of the sphere consisting of one bulk PIB chain, and the contour length of a PIB chain. On the other hand, the thicknesses of the hydrophilic PEG layers, (*d–D*), increase with the tetraPEG star size for two reasons. One is the larger size of the constituting tetraPEG stars, and the other is the greater aqueous swelling of the APCN hydrogels. Again, all (*d–D*) values expectedly fall within their minimum and maximum theoretical limits, also listed in the table, and calculated as the diameter of a bulk tetraPEG star and the contour length of a linear chain comprising two tetraPEG star arms, respectively.

## Conclusions

We have presented the development of a **model** amphiphilic hydrogel (APCN) system based on a *rare combination* of **highly incompatible**, and rather **large in size**, **well-defined polymer segments**, conferring to the system enhanced materials properties, both mechanical and morphological. Regarding advanced mechanical properties, these include a remarkable ***tensile strain at break***, reaching ***810%*** (at an APCN water content of 77%), and a noticeable ***tensile stress at break*** of ***215 kPa*** (64% water content). Regarding morphological properties, ***nanophase separation*** was observed both in ***D*_2_ *O*** and in the **bulk**, with the APCN samples based on the two larger tetraPEG stars exhibiting in D_2_O ***lamellae with long-range ordering***. We believe that this work lays the foundation for the emergence of APCNs as mechanically robust materials, despite their high water content, with a highly ordered nanostructure, and offering themselves for new applications in technology and biomedicine.

## Supplementary Material



## Notes

Chemical reactions leading to the preparation of α,ω-dibenzaldehyde-polyisobutylene (Bz–PIB–Bz); ^1^H NMR spectra, gel permeation chromatography (GPC) traces, and molar masses of the end-functionalized polyisobutylenes; tetraPEG star molar mass dependence of the 2nd-order reaction rate constant for the acylhydrazone group (cross-link) formation; tetraPEG star molar mass dependence of the APCN tensile mechanical properties; tensile stress–strain curves for annealed sample PIB–TetraPEG-20k; tetraPEG star molar mass dependence of the APCN domain sizes in D_2_O and in the bulk; tetraPEG star molar mass dependence of the hydrophobic and hydrophilic lamellar layer thicknesses; and details of the molecular thermodynamic model employed for determining the APCN morphologies in D_2_O (PDF)

## Notes

This work is dedicated to Prof. T. Alan Hatton of the Massachusetts Institute of Technology, on the occasion of his 75th birthday.
