# Supplementary material for: Synthesis of Highly Ordered Amphiphilic Polymer Conetwork Hydrogels via the Topologically Precise Interconnection of Two Highly Incompatible Polymers
Source: Chem Mater. 2025 Jul 24;37(15):5515–28. doi: 10.1021/acs.chemmater.5c00127 (PMC12356109; doi:10.1021/acs.chemmater.5c00127)
Supplement: Supplementary file 1 [file cm5c00127_si_001.pdf]

Supporting Information

Synthesis of Highly Ordered Amphiphilic Polymer Conetwork Hydrogels *via* the Topologically Precise Interconnection of Two Highly-Incompatible Polymers

by Demetris E. Apostolides,[1] George Michael,[1] Konstantinos Andronikou,[1] Costas S. Patrickios,[1,*] Szabolcs Pásztor,[2] Györgyi Szarka,[2] Anna Petróczy,[2] Béla Iván,[2] Takamasa Sakai,[3] Sylvain Prévost,[4] Dimitrios G. Tsalikis[5] and Michael Gradzielski[6]

Submitted for publication in *Chemistry of Materials*

January 2025

Revised: May and June 2025

[1] Department of Chemistry, University of Cyprus, P. O. Box 20537, 1678 Nicosia, Cyprus

[2] Polymer Chemistry and Physics Research Group, Institute of Materials and Environmental Chemistry, Hungarian Research Network, Research Centre for Natural Sciences, H-1117 Budapest, Magyar tudósok körútja 2, Hungary

[3] Department of Bioengineering, Graduate School of Engineering, The University of Tokyo, 7-3-1 Hongo, Bunkyo-ku, Tokyo 113-8656, Japan

[4] Institut Max von Laue - Paul Langevin (ILL), 71, avenue des Martyrs - CS 20156, 38042 Grenoble, cedex 9, France

[5] Particle Technology Laboratory, Department of Mechanical and Process Engineering, ETH Zurich, CH 8092, Switzerland

[6] Stranski-Laboratorium für Physikalische und Theoretische Chemie, Institut für Chemie, Straße des 17. Juni 124, Technische Universität Berlin, D-10623 Berlin, Germany

* Correspondence to: Costas S. Patrickios, **e-mail:** costasp@ucy.ac.cy

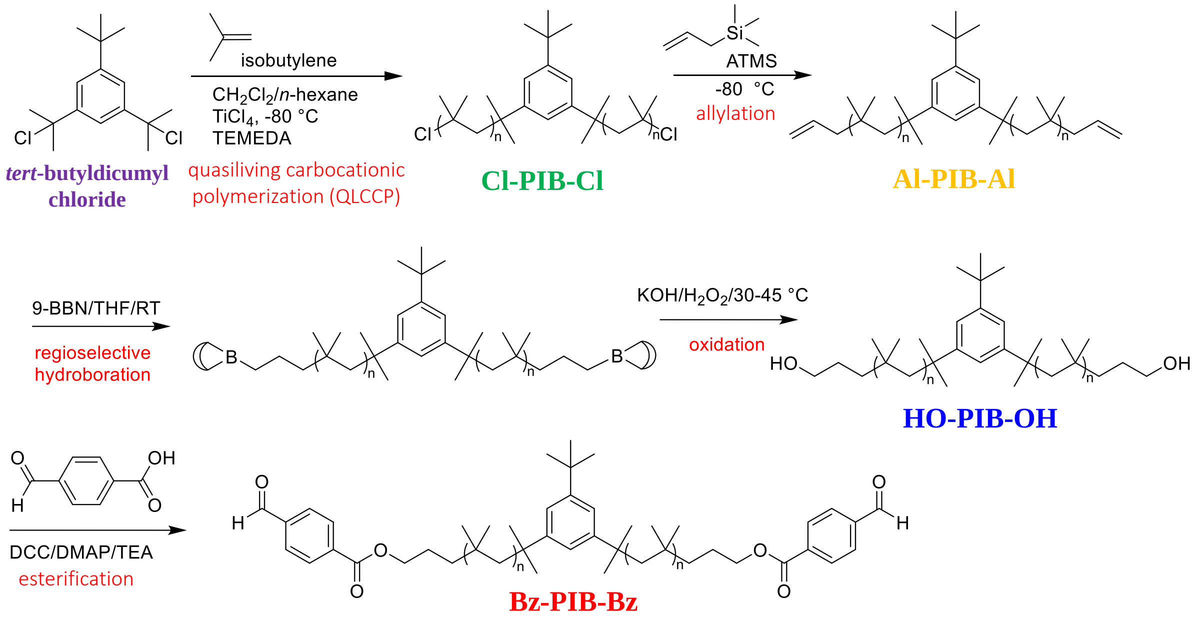

Figure S1. Synthesis of the hydrophobic building block, polyisobutylene (PIB), bearing two benzaldehyde (Bz) terminal groups (Bz-PIB-Bz) rendering it reactive to the hydrophilic tetraPEG star tetrabenzaacylhydrazide cross-linker. The synthesis is accomplished by the quasi-living carbocationic polymerization (QLCCP) of isobutylene using the bifunctional tert-butyldicumyl chloride initiator, together with $TiCl_4$/tetramethyleneethylenediamine (TEMEDA) in a dichloromethane/*n*-hexane mixture at –80 °C, followed by four quantitative end-group modification reactions, namely, allylation, regioselective hydroboration, oxidation and attachment of two benzaldehyde groups *via* esterification.

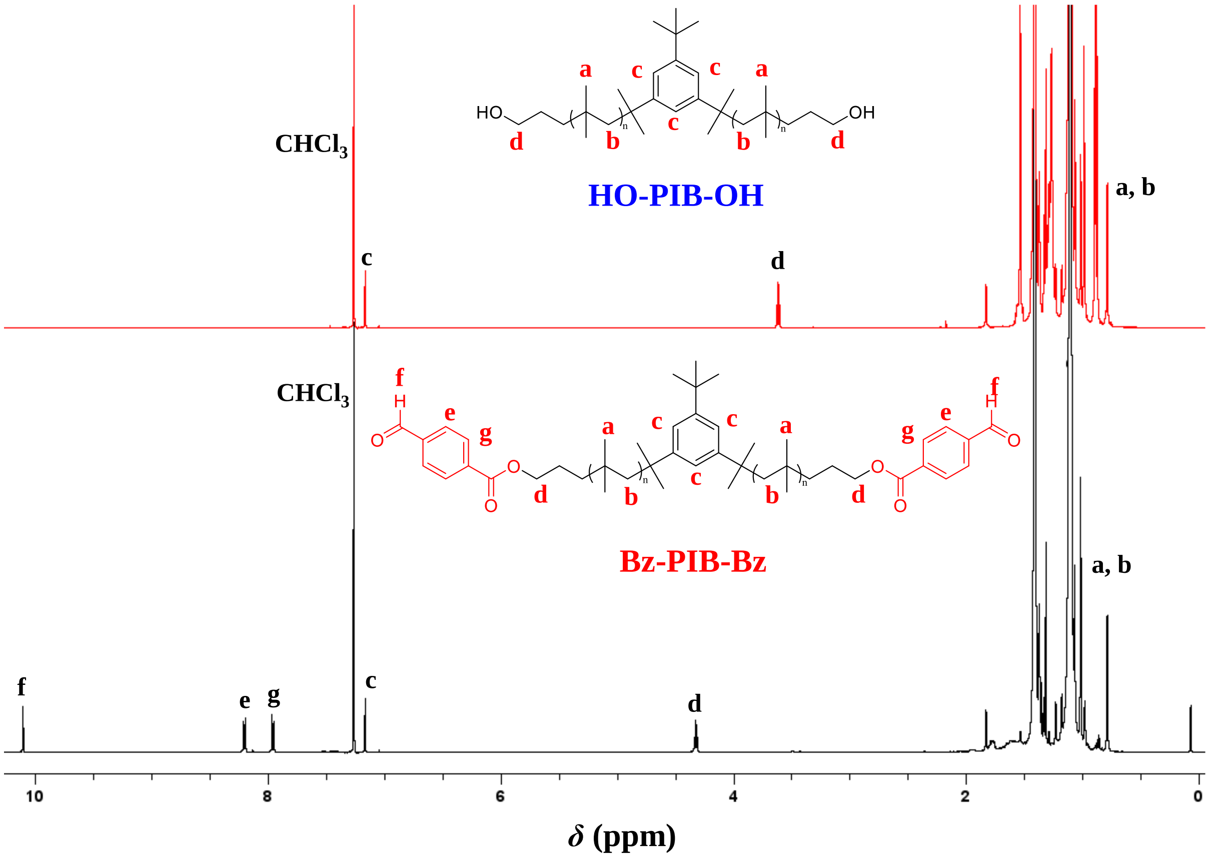

Figure S2. ^{1}H NMR (500 MHz) spectra in $CDCl_3$ for Bz-PIB-Bz and its HO-PIB-OH precursor. The spectra indicate quantitative conversion of the latter to the former.

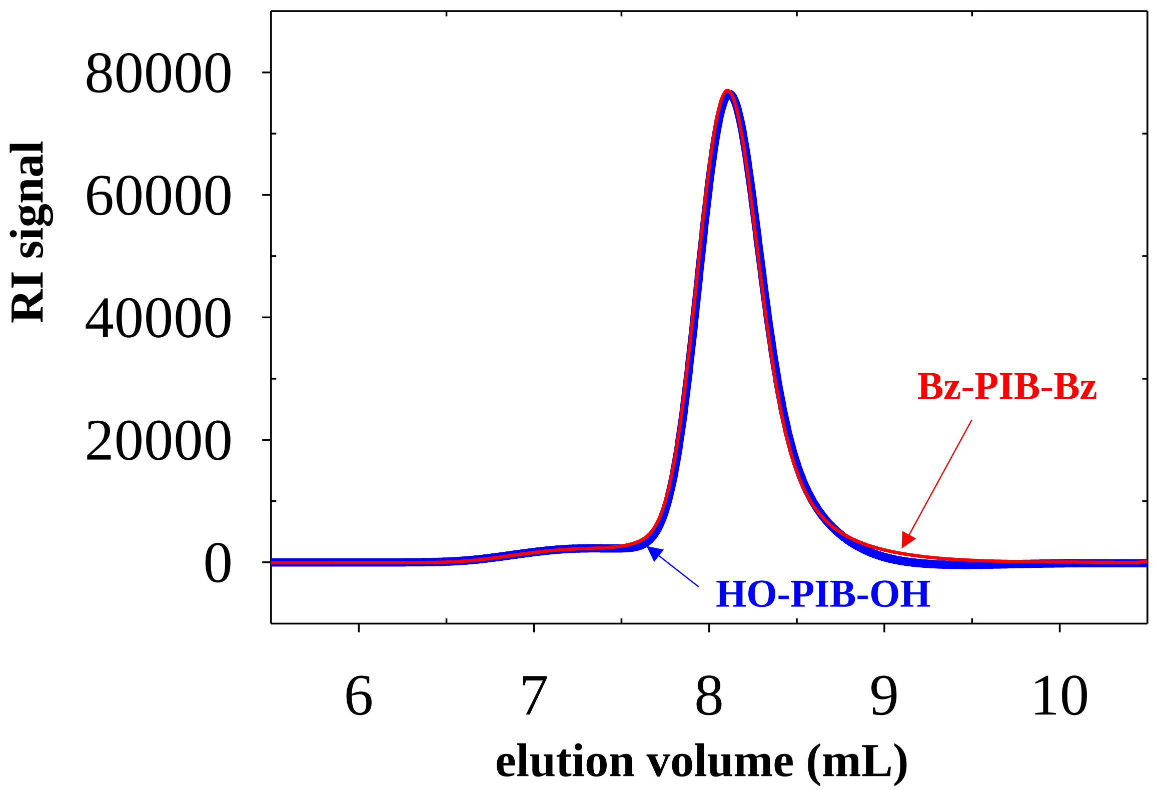

Figure S3. GPC traces for Bz-PIB-Bz and its HO-PIB-OH precursor. The mobile phase was THF delivered at 1 mL min^{-1} using an isocratic pump.

Table S1. Molar mass characteristics from GPC and ^{1}H NMR spectroscopy for Bz-PIB-Bz and HO-PIB-OH.

**Sample**		**Gel Permeation Chromatography (GPC)** [1]		**^{1}H NMR** [2]
		**M_n** (g mol^{-1})	***Đ***	**M_n** (g mol^{-1})
**HO-PIB-OH**		14 300	1.30	---
**Bz-PIB-Bz**		14 500	1.32	10 000

[1] Calibration based on linear, near-monodisperse poly(methyl methacrylate) standards.
[2] Analysis based on the benzaldehyde end-groups.

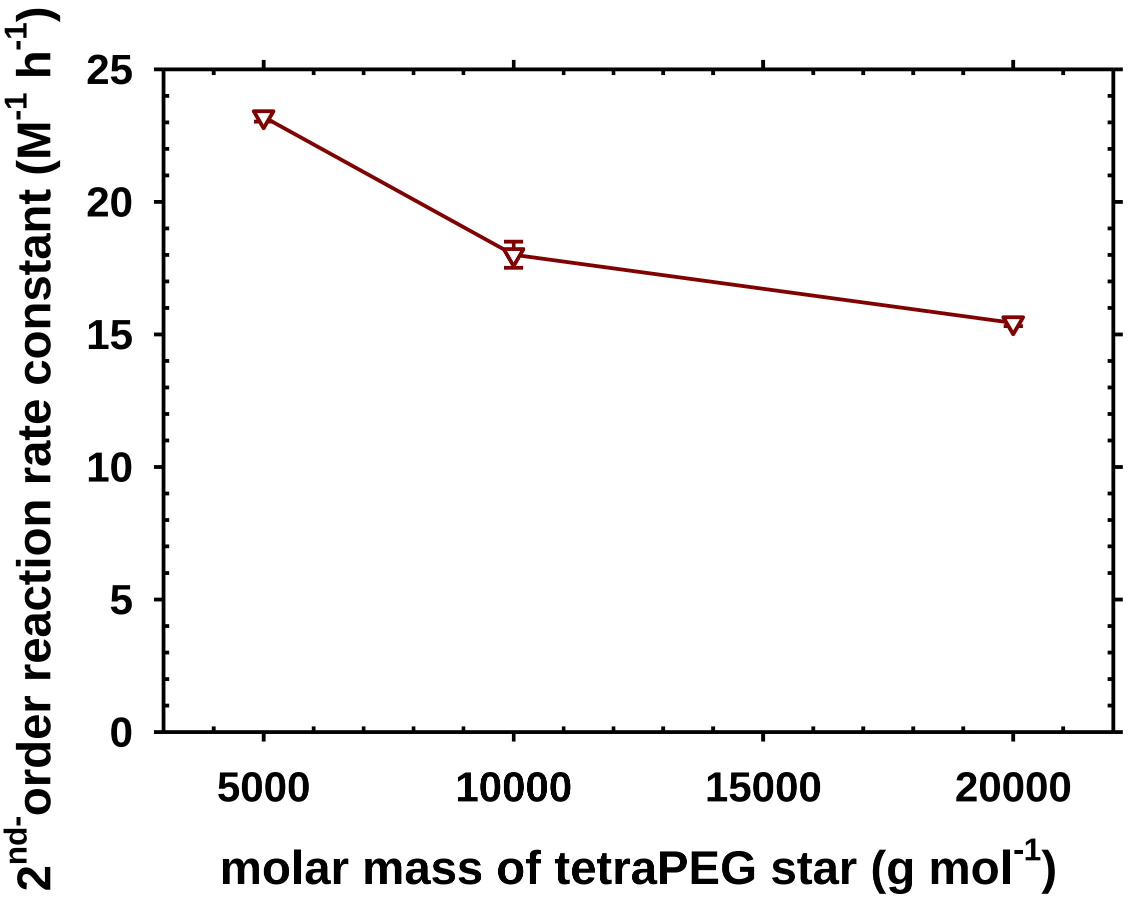

Figure S4. Dependence of the second-order reaction rate constant for the acylhydrazone group (cross-link) formation in benzene-d_6 on the molar masses of the constituting tetraPEG stars. The error bars represent ± 1 standard deviation in the value of the calculated rate constant, as that was estimated from the error propagated from the calculation of the slope in the linearized second-order plot in Figure 4(b) in the main text. All data are from Table 1 in the main text.

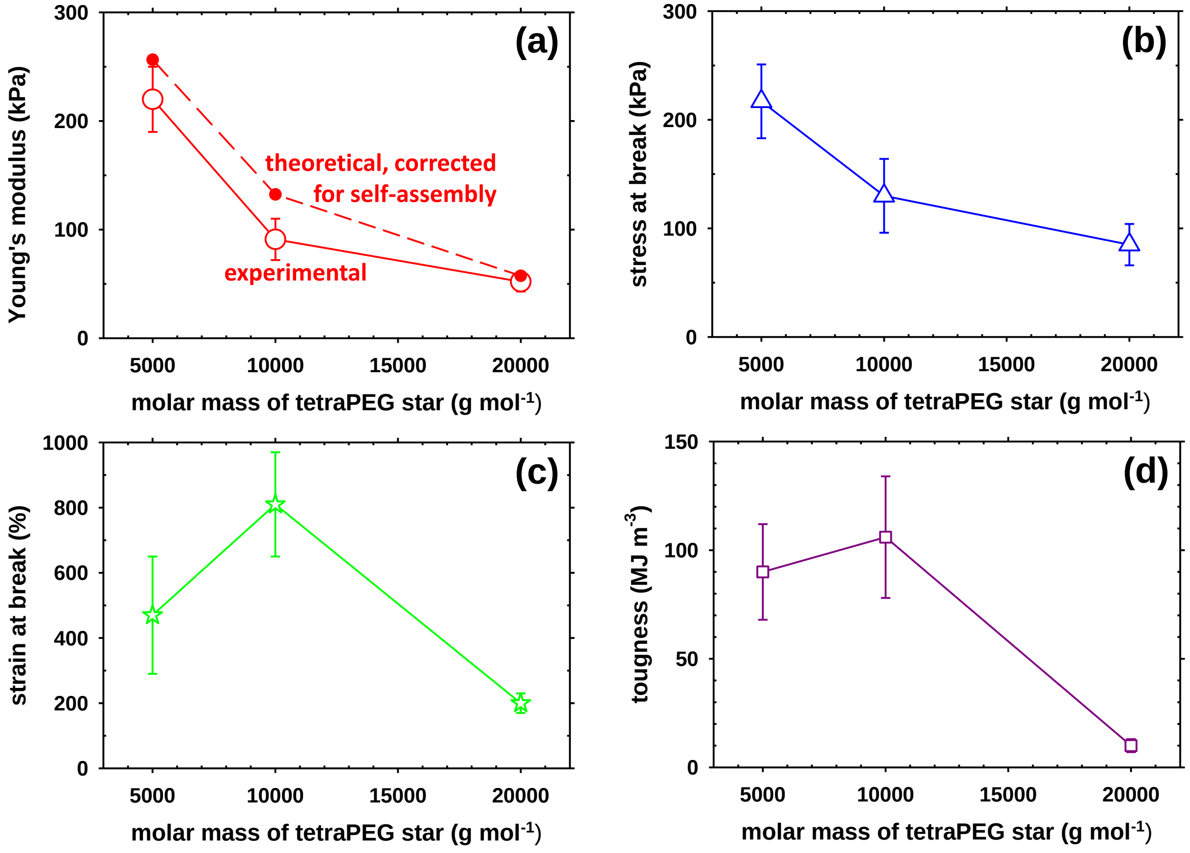

Figure S5. Dependence of the experimental and theoretical tensile mechanical properties of the three APCNs in water on the molar masses of the constituting tetraPEG stars. (a) Young's modulus. The theoretical Young's modulus was calculated as twice the value provided by the phantom network model, with the doubling representing the correction necessary to take into account the self-assembly of the PEG-PIB-PEG triblock chains in the aqueous milieu. (b) Stress at break. (c) Strain at break. (d) Toughness. The error bars in all the plots in the figure represent ± 1 standard deviation from the measurement. All data are from Table 2 in the main text.

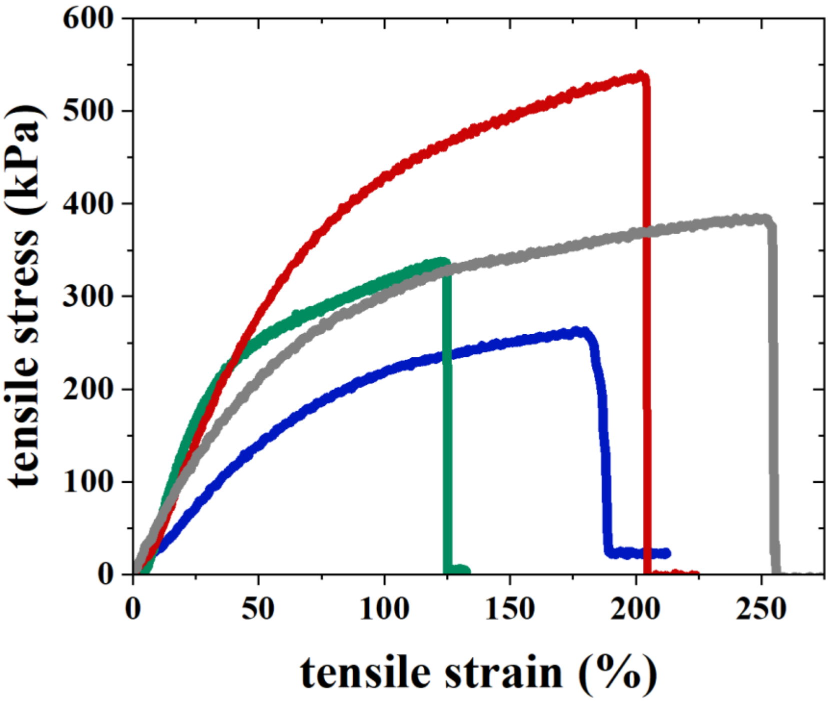

Figure S6. Tensile stress-strain curves (all four repetitions) for the thermally annealed PIB – TetraPEG-20k APCN in water at room temperature. Sample dimensions were 10 mm (length between clamps) × 4.0 mm (width) × 1.6 mm (thickness), whereas the elongation rate was kept at 300% min^{-1}.

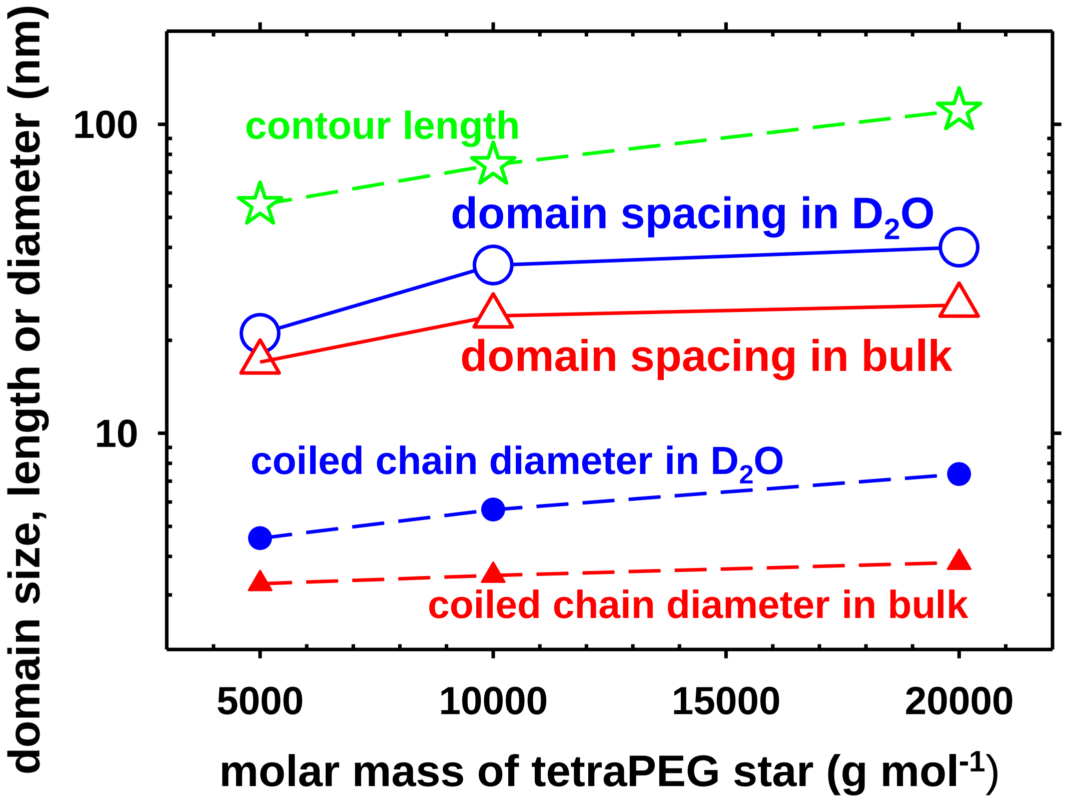

Figure S7. Variation of the domain spacings, *d*, in D_2O (open blue circles) and in the bulk (open red triangles) with the constituting tetraPEG star molar masses. All spacings were calculated from the *q*-position of the main peak in the SANS profiles, q_{max}, as $d = 2\pi/q_{max}$. These domain spacings expectedly have values intermediate between the corresponding minimum (closed blue circles and closed red triangles) and maximum (open green stars) spacings (also illustrated in the figure) estimated as the spherical coiled chain diameters and contour lengths, respectively. All data are from Table 3 in the main text.

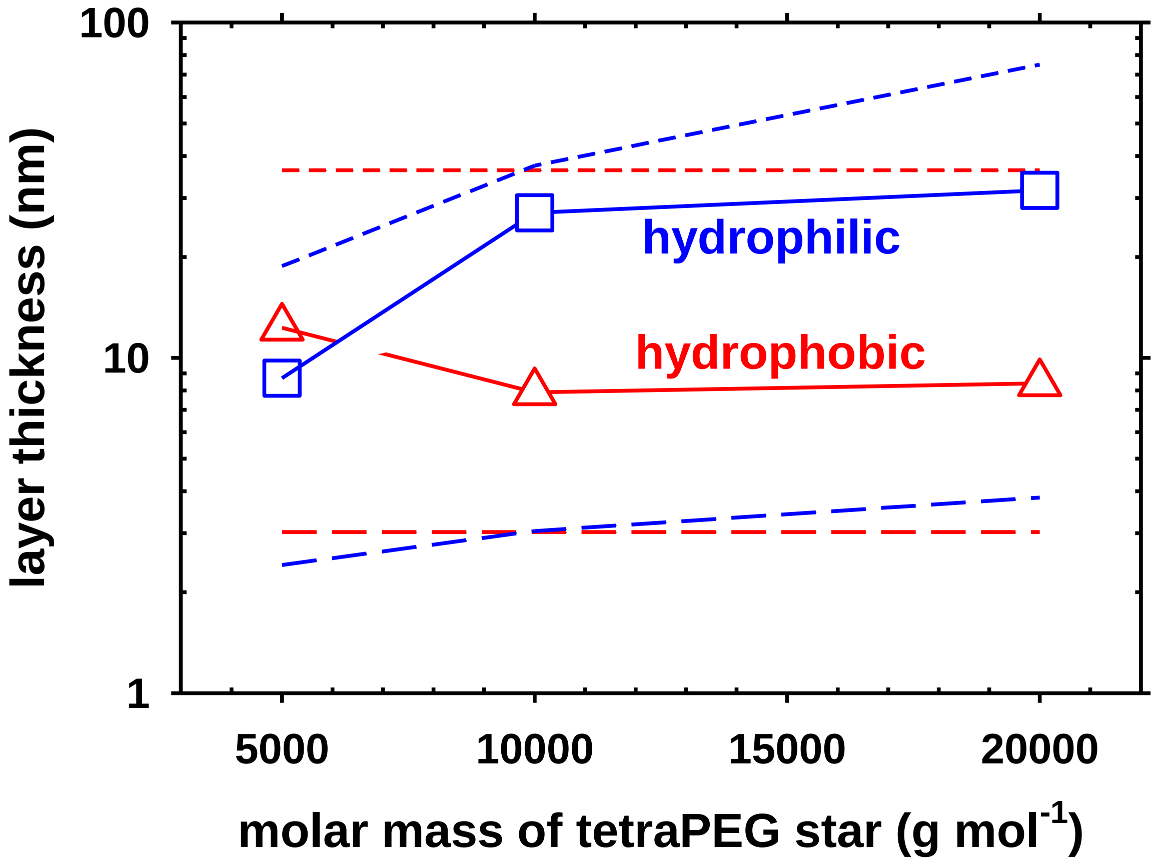

Figure S8. Variation of the hydrophobic (open red triangles) and hydrophilic (open blue squares) lamellar layer thicknesses with the constituting tetraPEG star molar masses. The hydrophobic layer thicknesses were calculated from the slopes in the Kratky plots in the intermediate-*q* regime (see Figure 9), whereas the hydrophilic layer thicknesses were determined by subtraction of the hydrophobic layer thicknesses from the corresponding domain spacings obtained from the primary SANS peaks. The hydrophobic and hydrophilic layer thicknesses are expectedly sandwiched between the corresponding minimum (long dashed lines) and maximum (shorter dashed lines) thickness values (also illustrated in the figure) estimated as the anhydrous spherical coil diameter and contour length of the corresponding polymer segment, respectively. All data are from Table 4 in the main text.

Molecular Thermodynamic Model

A molecular thermodynamic model[1-3] was employed to confirm the lamellar morphologies identified from the SANS profiles of the two APCNs containing the two larger tetraPEG stars, and to elucidate the morphology of the third APCN containing the smallest tetraPEG star. This model considers four possible states for the APCNs: the disordered state and the three main ordered morphologies, the lamellar, the cylindrical and the spherical. For the disordered state, the system was described by the mixing Gibbs free energy, comprising an entropic and an enthalpic component, and the elastic Gibbs free energy. For the ordered morphologies, in addition to the mixing and elastic Gibbs free energy components, another Gibbs free energy component was necessary to be introduced so as to account for the creation of an interface between the two different nanophases formed: the **interfacial** Gibbs free energy, that captures the cost for maintaining necessary boundaries between the hydrophilic and hydrophobic nanodomains. All the above Gibbs free energy components are functions of various system parameters that are named and defined in **Table S2**, where their chosen values are also given.

Table S2. Parameters used in constructing the molecular thermodynamic model for the aqueous APCN systems.

Parameter	Definition	Value
f	Crosslinker functionality (number of arms emanating out of each crosslink)	4
N	Degree of polymerization of the half-chain (chain in the whole unit cell)	100, 120 and 160*
η	Number of monomer repeating units in the hydrophilic block in the whole unit cell	20, 40 and 80*
ζ	Number of monomer repeating units in the hydrophobic half-block in the whole unit cell	80
χ_A	Flory-Huggins interaction parameter between the hydrophilic monomer repeating units and water	0.0
χ_B	Flory-Huggins interaction parameter between the hydrophobic monomer repeating units and water	2.0
χ_{AB}	Flory-Huggins interaction parameter between the hydrophilic and hydrophobic monomer repeating units	2.0

* These values correspond to the APCNs containing the TetraPEG-5k, TetraPEG-10k and TetraPEG-20k star components.

By minimizing, using MATLAB,[4] the total Gibbs free energy with respect to the polymer volume fraction for each of the four states, *i.e.*, the three ordered morphologies and the disordered state, to find $\Delta G_{\text{total,min}}$ for each state, one would choose the state with the lowest $\Delta G_{\text{total,min}}$ value, which would correspond to the prevailing morphology. This was done for all three APCNs possessing different hydrophilic polymer volume fractions (= η/N) of 0.20, 0.33 and 0.50. The calculated $\Delta G_{\text{total,min}}$ values for each of the four states for each of the three APCNs are listed in **Table S3**, where one may observe that the prevailing morphology was the lamellar one for all three APCNs. This is consistent with previous calculations[2,3] that also indicated that lamellae would prevail for APCNs in water with compositions the same as the ones in the present work, but different chain architecture.

Table S3. Values of the minimized total Gibbs free energy for the four possible states of the three APCNs studied.

	$\Delta G_{\mathbf{total,min}}$		
**Morphology**	***TetraPEG-5k***	***TetraPEG-10k***	***TetraPEG-20k***
Disordered	-39.252	-65.899	-138.827
Spherical	3.728	-2.290	-34.912
Cylindrical	-7.447	-27.680	-88.739
Lamellar	**-66.263**	**-126.627**	**-253.300**

References

[1] Vamvakaki, M.; Patrickios, C. S. Polyelectrolytic Amphiphilic Model Networks in Water: A Molecular Thermodynamic Theory for Their Microphase Separation. *J. Phys. Chem. B* **2001**, *105*, 4979-4986.

[2] Georgiou, T. K.; Vamvakaki, M.; Patrickios, C. S. Georgiou, T. K.; Vamvakaki, M.; Patrickios, C. S. Microphase Separation Under Constraints: A Molecular Thermodynamic Theory for Polyelectrolytic Amphiphilic Model Networks in Water, *Polymer* **2004**, *45*, 7341-7355.

[3] Varnava, C. K.; Patrickios, C. S. Model Amphiphilic Polymer Conetworks in Water: Prediction of Their Ability for Oil Solubilization. *ACS Omega* **2019**, *4*, 4721-4738.

[4] Hunt, B. R.; Lipsman, R. L.; Rosenberg, J. M. *A Guide to MATLAB for Beginners and Experienced Users*, Cambridge University Press, Cambridge, UK, 2001.
